# Methionine and Tryptophan Play Different Modulatory Roles in the European Seabass (*Dicentrarchus labrax*) Innate Immune Response and Apoptosis Signaling—An *In Vitro* Study

**DOI:** 10.3389/fimmu.2021.660448

**Published:** 2021-03-15

**Authors:** Marina Machado, Cláudia R. Serra, Aires Oliva-Teles, Benjamín Costas

**Affiliations:** ^1^ Centro Interdisciplinar de Investigação Marinha e Ambiental (CIIMAR), Matosinhos, Portugal; ^2^ Instituto de Investigação e Inovação em Saúde (i3S), Universidade do Porto, Porto, Portugal; ^3^ Instituto de Ciências Biomédicas Abel Salazar (ICBAS-UP), Universidade do Porto, Porto, Portugal; ^4^ Instituto de Biologia Molecular e Celular, Universidade do Porto, Porto, Portugal; ^5^ Departamento de Biologia, Faculdade de Ciências da Universidade do Porto (FCUP), Porto, Portugal

**Keywords:** amino acids, inflammation, fish, AIP56, *Photobacterium damselae* subsp. *piscicida*

## Abstract

The range of metabolic pathways that are dependent on a proper supply of specific amino acids (AA) unveils their importance in the support of health. AA play central roles in key pathways vital for immune support and individual AA supplementation has shown to be able to modulate fish immunity. *In vitro* trials are important tools to evaluate the immunomodulatory role of AA, and the present study was conceived to evaluate methionine and tryptophan roles in immune-related mechanisms aiming to understand their effects in leucocyte functioning and AA pathways. For that purpose, head-kidney leucocytes were isolated and a primary cell culture established. The effect of methionine or tryptophan surplus on cell viability was assessed. Medium L-15 10% FBS without AA addition (0.5mM of L-methionine, 0.1 mM of L-tryptophan) was used as control. To that, L-methionine or L-tryptophan were supplemented at 1 and 2 times (M1x or M2x, and T1x or T2x). Nitric oxide, ATP, total antioxidant capacity, and immune-related genes were evaluated in response to lipopolysaccharides extracted from *Photobacterium damselae* subsp. *piscicida* or UV-inactivated bacteria). Moreover, caspase 3 activity and apoptosis-related genes were evaluated in response to the apoptosis-inducing protein, AIP56. Distinct roles in leucocytes’ immune response were observed, with contrasting outcomes in the modulation of individual pathways. Methionine surplus improved cell viability, polyamine production, and methionine-related genes expression in response to an inflammatory agent. Also, methionine supplementation lowered signals of apoptosis by AIP56, presenting lower caspase 3 activity and higher *il1β* and *nf-κb* expression. Cells cultured in tryptophan supplemented medium presented signals of an attenuated inflammatory response, with decreased ATP and enhanced expression of anti-inflammatory and catabolism-related genes in macrophages. In response to AIP56, leucocytes cultured in a tryptophan-rich medium presented lower resilience to the toxin, higher caspase 3 activity and expression of caspase 8, and lower expression of several genes, including *nf-κb* and *p65*. This study showed the ability of methionine surplus to improve leucocytes’ response to an inflammatory agent and to lower signals of apoptosis by AIP56 induction, while tryptophan attenuated several cellular signals of the inflammatory response to UV-inactivated bacteria and lowered leucocyte resilience to AIP56.

## Introduction

Amino acids (AA) are key players for the biosynthesis of vital molecules for immune support (1) and their use as nutraceutical supplements in mammals (2) and poultry (3, 4) is a current strategy. The role of individual AA in the innate immune response is associated with their role in key pathways essential for cellular function and as precursors of hormones and enzymes. Likewise, following the presuppose that the requirement of specific AA increases in response to inflammation and infection (5), their dietary supplementation may allow the modulation of particular immune-related pathways with the final goal of enhancing host immunity. Having this in mind, *in vivo* studies were developed to achieve nutritional strategies that modulate the fish immune response through the increase of AA availability (6–14), while few *in vitro* studies (15, 16) aiming to collect information at the cellular level were performed.

Previous studies in fish contributed with some insights on the key role of methionine in the fish immune system. Dietary methionine supplementation was able to increase European seabass (*Dicentrarchus labrax*) peripheral neutrophil numbers and innate immune response to an inflammatory insult with UV-inactivated *Photobacterium damselae* subsp. *piscicida* (*Phdp*) (11, 13) and increase disease resistance to *Phdp* (14). Also, juvenile Jian carp (*Cyprinus carpio* var. Jian) fed with increased levels of methionine presented an increased peripheral leucocyte concentration and humoral response, in response to *Aeromonas hydrophila* (9). Additionally, methionine supplementation was shown to be key for European seabass immune support in an extreme feed formulation (0% fish meal) (17). Data from those studies are sustained by the recognized role of methionine as a methyl group donor for the methylation, transsulfuration, and aminopropylation routes, with coenzyme S-adenosylmethionine (SAM) as the leading factor. The increase of methionine input can increase DNA methylation ratio, known to be dependent on the supply of SAM (18). Through the aminopropylation route, methionine contributes to polyamine synthesis thus contributing to cell proliferation (19). Finally, by the transsulfuration pathway, methionine is the precursor of cysteine, an AA constituent of the powerful antioxidant molecule glutathione (GSH) (1). *In vitro* results on this matter are scarce. Nonetheless, Azeredo et al. (16) showed the ability of methionine supplementation to boost European seabass head-kidney (HK) leucocytes (HKL) nitric oxide (NO), superoxide anion, and ATP production in the absence of stimulus, and improved HKL NO production in response to UV-inactivated *Vibrio anguillarum*. These authors discussed that methionine potential for immune improvement seems to be directly related to the enhancement of cell response by the enhancement of the methionine-related pathways.

Tryptophan has been the target of *in vivo* studies in several animal models focusing its function as a precursor of compounds involved in stress modulation, antioxidant properties, and immune tolerance (20–22). Studies in fish showed that increasing dietary tryptophan levels did not significantly modulate European seabass (11) and Persian sturgeon (*Acipenser persicus*) (23) basal innate immune- related mechanisms activity, while a clear effect of tryptophan surplus was observed upon inflammation induction. For instance, in European seabass Azeredo et al. (12) observed an inhibitory action of tryptophan on HK inflammatory transcripts while Machado et al. (24) reported a compromised immune response and disease resistance to *Phdp*. In macrophages, tryptophan catabolism occurs through the kynurenine-niacin pathway, mediated by indoleamine 2, 3-dioxygenase (IDO) (25). In response to inflammation, IDO is induced, exerting anti-microbial effects by tryptophan extracellular depletion (26, 27), setting an antioxidant system by the consumption of superoxide radicals, and its metabolites regulate T-cell function (28). *In vitro* results pointed to the impairment of pro-inflammatory signals offsetting the inflammatory response caused by tryptophan higher availability (16).

The inflammatory response and the overall innate immune system is sustained by phagocytes (i.e. neutrophils and macrophages). These myeloid cells are responsible for the incorporation and digestion of other cells or cell components, as pathogens or apoptotic and necrotic host-cells. Moreover, fish phagocytes are responsible for the initiation and resolution of inflammation by the production and secretion of pro- and anti-inflammatory chemical messengers (autocrine signaling) and cell-to-cell signaling communication (paracrine signaling) (29). In fact, as widely described in mammals, monocytes can adapt their phenotypes according to the response stage and differentiated into three distinct groups formerly determined by the inductor of differentiation (30–33). Upon activation, circulating monocytes (M0 type) are differentiated into macrophages that under inflammatory settings and in the presence of pathogens and tumor cells, are described as M1 type. Whereas M2 macrophages are found in sterile conditions or in the repair stage of the innate response (34). This has only been recently described in fish, particularly in the common carp (*Cyprinus carpio* L) (35). Additionally, cell inactivation and clearance through apoptosis takes part in the proper function of the immune system. As a defense mechanism, apoptosis is responsible for the clearance of damaged cells and can be initiated by a wide variety of stimuli, as infection and stress (36). Since macrophages present themselves as a useful tool for *in vitro* functional studies on the fish innate immune response, the present work focused on the study of the response of innate activated macrophages induced by microbial stimuli and with key roles in the phagocytosis of pathogens and the production of pro-inflammatory signals (37).

*Phdp* is a bacterial pathogen for many marine fish species that owns part its successful pathogenesis to the ability to produce and release the exotoxin AIP56 to the extracellular environment (38). AIP56 cleaves the transcription factor NF- κB, therefore inhibiting the transcription of key immune-related genes (39) and inducing extensive apoptosis of macrophages and neutrophils (38). Therefore, *Phdp* can act at distance, without direct contact with the host cells, and leads to the depletion of host primary cell defense mechanisms, the phagocytes.

The present study aimed to evaluate *in vitro*, at the leucocytes functional and transcriptional level, the modulatory effects of two supplementation levels of methionine and tryptophan on the response of HKL against UV- inactivated *Phdp* (*Phdp*UV) or *Phdp* lipopolysaccharides (LPS*Phdp*), and the apoptosis process in response to the AIP56 toxin.

## Material and Methods

The experiments were approved by the CIIMAR Animal Welfare Committee, carried out in a registered installation (N16091.UDER), and performed by trained scientists in full compliance with national rules and following the European Directive 2010/63/EU of the European Parliament and the European Union Council on the protection of animals used for scientific purposes.

### *Photobacterium damselae subsp. piscicida* Inactivation

*Phdp* strain PP3, isolated from yellowtail (*Seriola quinqueradiata*; Japan), was obtained from the Fish Immunology and Vaccinology group (i3S/IBMC, University of Porto). Bacteria were routinely cultured at 22°C in tryptic soy broth (TSB) or tryptic soy agar (TSA) (both from Difco Laboratories) supplemented with NaCl to a final concentration of 2% (w/v) (TSB-2 and TSA-2, respectively). Live bacteria were stored at −80°C in TSB-2 supplemented with 15% (v/v) glycerol.

To maintain the structural integrity of bacterial antigens but prevent bacterial growth, *Phdp* were killed by UV irradiation. For this, bacteria were grown in TSB-2 and, after reaching the exponential growth phase (OD_600nm_= 0.523; 6.7 x 10^8^ colony forming units (CFUs) ml^-1^), were placed in a sterile tray (maximum inoculum height of 0.2 mm) at a distance of 10 centimeters of a UV lamp and inactivated by exposure to UV irradiation for 2 h. Bacteria were then recovered by centrifugation at 1500 × *g* for 30 min, the pellet resuspended in Hank’s Balanced Salt Solution (HBSS, Gibco), and bacterial concentration adjusted to a virtual dose of 1 × 10^7^ CFU ml^-1^ taking into account their concentration prior to inactivation. Lack of bacterial viability was confirmed by plating the inoculum on TSA-2.

### *Photobacterium damselae subsp. piscicida* Lipopolysaccharides Extraction and Purification

Lipopolysaccharides (LPS) from *Phdp* (LPS*Phdp*) were extracted by hot phenol-water according to the method described by Rezania et al. (40) with modifications (16). Purified LPS*Phdp*, without any residual phenol, was lyophilized, resuspended in PBS to a final concentration of 2 mg ml^−1^, and kept at −20°C until use. Visualization was achieved by SDS-PAGE (12%) electrophoretic resolution of 20 µg purified LPS*Phdp* and consequent staining following the improved silver stain protocol described by Zhu et al. (41).

### Recombinant AIP56

Recombinant AIP56 (38) was obtained from the Fish Immunology and Vaccinology group (i3S/IBMC, University of Porto) and stored in 20 nM Tris-HCl, pH 8.0 at -80 °C until use.

### Fish and Establishment of HKL Primary Cell Cultures

European seabass (*Dicentrarchus labrax*) juveniles (8 ± 0.5 g) were obtained from a certified hatchery (MARESA, Spain), acclimatized to laboratory conditions, and reared for 2 years in a recirculation water system to a final body weight of 700 ± 50 g. Water parameters were maintained as followed: O_2_ saturation at 7.38 ± 0.01 mg/l, salinity at 35 ppt, temperature at 18°C, and 10 h dark: 14 h light photoperiod. Fish were daily fed a commercial diet (Sorgal, Portugal) and no clinical signs of disease and illness were observed.

European seabass were euthanized by an overdose of anesthetic (>1ml/l, 2-phenoxyethanol, Merck) and bled by collecting blood from the caudal vessels using a heparinized vacuum system and cut off the branchial arches. HKL were isolated and maintained following Secombes (42) with modifications. The head-kidney was aseptically removed and the tissue disrupted by passing through a 100 µl nylon mesh in 30 mL of Leibovitz L-15 medium (L-15, Gibco) supplemented with 2% fetal bovine serum (FBS, Gibco), 100 IU ml^-1^ penicillin and streptomycin (Gibco), and 30 U ml^-1^ heparin (Braun) (L-15 2% FBS). After centrifugation, a clear separation between leucocytes and erythrocytes was observed and the upper leucocyte layer was carefully collected and separated from the erythrocytes bottom layer and the resulting cell suspension was resuspended in fresh L-15 2% FBS and centrifuged at 600 × *g* at 4°C for 10 minutes. The collected leucocytes were washed two times at 600 × *g* at 4°C for 10 minutes and finally resuspended in L-15 medium with 0.1% FBS (L-15 0.1% FBS) and antibiotics. Leucocytes viability was determined by the trypan blue exclusion method. Leucocytes were diluted four times in a 0.4% trypan blue solution and concentration adjusted to 1 × 10^7^ viable cells ml^-1^ after counting in a hemocytometer. One hundred microliters of the leucocyte suspension were plated in 96-well plate for ATP, total antioxidants concentration (TAC), polyamines, nitric oxide (NO), and caspase 3-active assays. For gene expression, 500 µl of the leucocyte suspension was plated in 24-well plates.

### Experimental Design

After primary cell culture isolation and plating, the leucocyte monolayer was maintained for 2 h in L-15 2% FBS for cell adhesion and subsequently washed with HBSS, removing non-adherent cells. Adherent cells, characterized mostly by the monocyte lineage (43) were then incubated with fresh L-15 10% FBS supplemented with methionine or tryptophan for 24 h at 18 °C and leucocyte viability was evaluated by the 3-(4,5-dimethylthiazol-2-yl)-2, 5-diphenyltetrazolium bromide (MTT) assay. Two supplementation levels of each AA were selected based in previous studies (16). Both AA were supplemented at 1 × or 2 × the basal concentration found in L-15 with the final concentration of L-methionine (M1x, 1mM or M2x, 1.5 mM) and L-tryptophan (T1x, 0.2 mM or T2x, 0.3 mM). As a control, L-15 10% FBS without AA addition was used (containing 0.5mM of L-methionine and 0.1 mM of L-tryptophan, Gibco).

**Figure 1:**
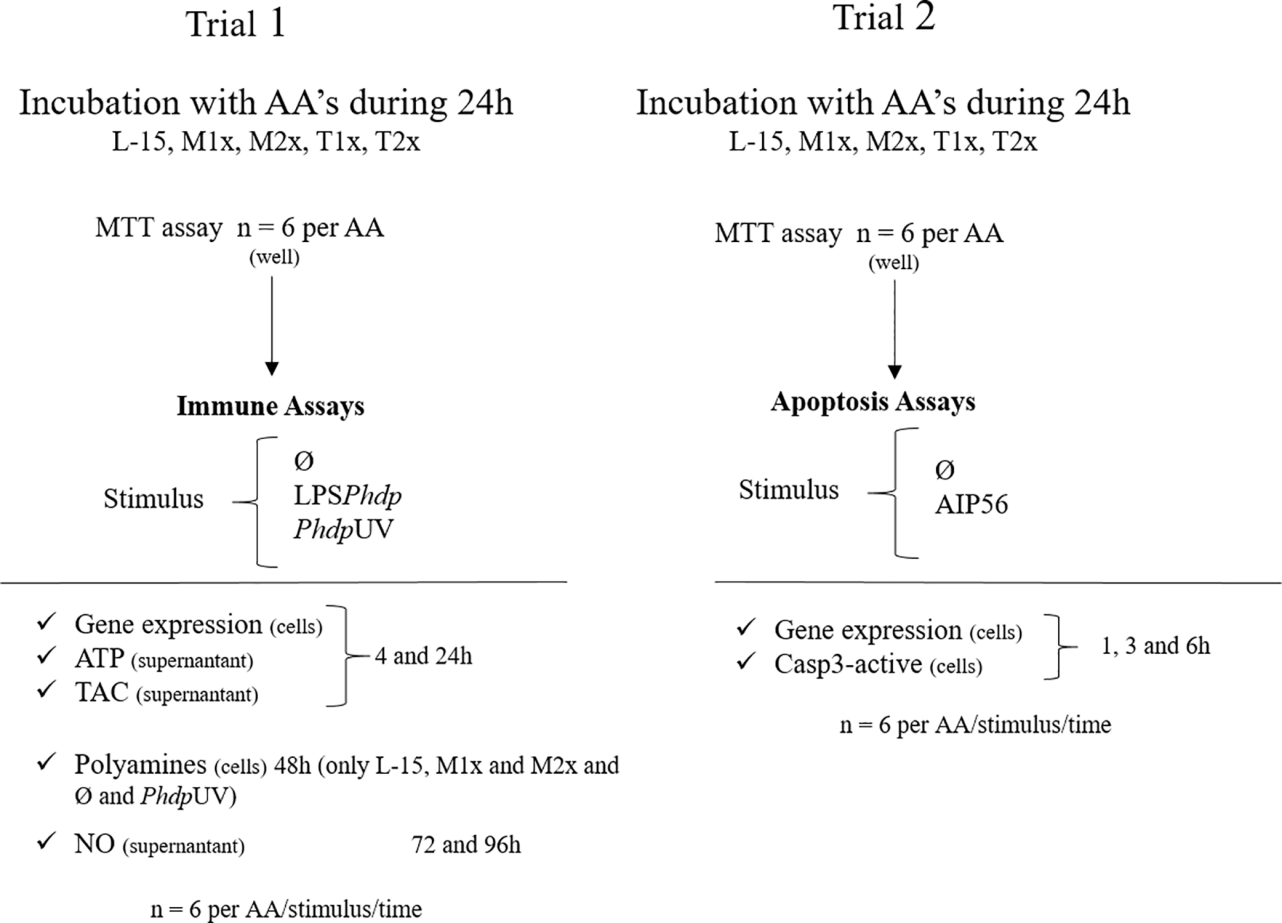
Experimental design. L-15 (Leibowitz L-15 medium); M1x (Leibowitz L-15 medium supplemented with 1 mM of L-methionine); M2x (Leibowitz L-15 medium supplemented with 1.5 mM of L-methionine); T1x (Leibowitz L-15 medium supplemented 0.2 mM of L-tryptophan); T2x (Leibowitz L-15 medium supplemented 0.3 mM of L-tryptophan); MTT (3-(4,5-dimethylthiazol-2-yl)-2, 5-diphenyltetrazolium bromide assay); Ø (unstimulated cells); LPS*Phdp* (Lipopolysaccharides extracted from *Phdp*); *Phdp*UV (UV-inactivated *Phdp*); ATP (Adenosine triphosphate); TAC (total antioxidant capacity); NO (nitric oxide).

Two trials, each comprising a total of six fish were performed (**Figure 1**). Cellular response (Trial 1) was assessed upon stimulation with *Phdp*UV (1 × 10^6^ CFU ml^-1^ with an expected LPS concentration of 0.006 µg ml^-1^), LPS*Phdp* (10 µg ml^-1^), or the absence of a stimulus (Ø). Concentration of each stimulus was selected according to (16). Gene expression, ATP, and TAC were measured at the end of 4 and 24 h of stimulation since most innate immune mechanisms are activated upon acute stimulation. Polyamines production was evaluated in L-15, M1x, and M2x after 48 h of stimulation since methionine has a direct role in polyamine biosynthesis (19), but only in *Phdp*UV and Ø treatments due to technical limitations. Finally, NO production was evaluated at 72 and 96 h according to previous studies (16, 44). The apoptosis assays (Trial 2) consisted of evaluating caspase 3-active and gene expression in response to AIP56 protein (2 µg ml^-1^) (45) or in Ø during a time-course study (i.e. 1, 3, and 6 h) with the cell culture media replaced by a fresh solution at each hour. Staurosporine (2.33 µg ml^-1^, Sigma) was used in L-15 wells as a positive control for apoptosis (46).

All analyses were performed with triplicate analytic replicates in a total of six biological replicates.

### MTT Assay

The leucocytes viability was assessed by the MTT reduction by the NAD(P)H-dependent cellular oxidoreductase enzymes produced under cell metabolic activity, reflecting the number of viable cells (47). After 24 h incubation at 18 °C in L-15 10% FBS supplemented with the desired AA, 20 µl of MTT (5 mg ml^-1^) was added to each well and incubated for 4 h at 18 °C. After centrifugation at 110 × *g* for 5 min, the insoluble resultant formazan was dissolved in 100 µl of dimethyl sulfoxide (DMSO) and the absorbance read at 550 nm (Synergy HT, Biotek). A total of twelve biological replicates were evaluated (6 biological replicates per trial).

### Immune Assays

#### ATP Assay

ATP production by the leucocytes monolayers incubated with LPS*Phdp*, *Phdp*UV, or in the absence of stimuli for 4 and 24 h with each AA treatment was measured with an ATP Colorimetric Assay Kit (Sigma). From each well, 50 µl of the cell supernatant was transferred to a new 96-well plate and the protocol performed according to the manufacture’s indications. The absorbance was read at 570 nm (Synergy HT, Biotek) and ATP concentration was calculated according to a standard ATP curve after subtraction of background absorbance values.

#### TAC Assay

The total antioxidants concentration (TAC), such us oxygen species and reactive nitrogen species, was determined in the supernatant of cells incubated with LPS*Phdp*, *Phdp*UV, or Ø during 4 and 24 h and previously incubated for 24 h with the different AA treatments. Cell supernatants were collected, diluted in Protein Mask in a 1:1 ratio according to manufacturer’s indications (The Total Antioxidant Capacity Kit, Sigma), and 100 µl of the mixture was added to a 96-well plate and the protocol performed. The absorbance was read at 570 nm (Synergy HT, Biotek). Background absorbance values were subtracted and TAC concentration was calculated from a standard curve.

#### Polyamines Assay

The Total Polyamine Assay Kit (BioVision) was used for fluorometric assessment of polyamine content in cells either stimulated with *Phdp*UV or unstimulated for 48 h after incubation with L-15, M1x, or M2x for 24 h. According to the manufacturer’s indications, a sample background wells fluorescence (Ex/Em= 535/587 nm) was subtracted to the standards and reaction wells and the concentration determined according to the standard curve previously prepared.

#### NO Assay

NO production was measured in the supernatant collected from leucocyte primary cell cultures formerly incubated with the different AA treatments for 24 h and stimulated with *Phdp*LPS, *Phdp*UV, or in the absence of stimuli for 72 or 96 h. At each time, 50 µl of supernatant was transferred to a new 96-well plate and total nitrite and nitrate concentrations in the sample were assessed using the Nitrite/Nitrate colorimetric method kit (Roche). Nitrite concentration was calculated by comparison with a sodium nitrite standard curve. Since nitrite and nitrate are endogenously produced as oxidative metabolites of the messenger molecule NO, these compounds are considered as indicative of NO production.

### Apoptosis Assays

After incubation for 24 h with the different AA treatments, the cell culture media was replaced by a fresh solution without stimuli or containing AIP56. The medium was renewed at each hourly, for 6 h (45), and cells collected for caspase 3-active assay or for gene expression after 1, 3 and 6 hours. The inability of the AA, at the highest supplementation level, to inhibit AIP56 activity on the cleavage of the leucocytes p65 subunit was tested by SDS-PAGE and Western blotting.

#### SDS-PAGE and Western Blotting

Leucocytes from four fish were incubated with L-15, M2x or T2x during 24h and afterwards incubated the with Ø or 2 µg ml^-1^ of AIP56 for 2 h. Supernatant was removed and cells (5× 10^6^ cells) were lysed and collected by adding 40 µl SDS-PAGE sample buffer (50mMTris-HCl [pH 8.8], 2% SDS, 0.05% bromophenol blue, 10% glycerol, 2mM EDTA, and 100 mM DTT) (48). Samples were then boiled for 5 min and 20 µl (2.5× 10^6^ cells) were loaded For Western blotting, the proteins were transferred onto nitrocellulose membranes. The efficiency of transfer and the protein loading on the membranes was controlled by staining with Ponceau S. The membranes were blocked for 1 h at room temperature (RT) with 5% skim milk in Tris-buffered saline (TBS) containing 0.1% Tween 20 (T-TBS) followed by incubation for 1 h at RT with the anti-sea bass NF- κB p65 rabbit serum diluted in blocking buffer (1:500) (49). Immunoreactive bands were detected using alkaline phosphatase-conjugated secondary antibodies (1:0000) and nitrobluetetrazolium–5-bromo-4-chloro-3-indolylphosphate (NBT/BCIP) (Promega).

#### Caspase 3-Active

The caspase 3-active assay was performed for each AA treatment in cells stimulated for 1, 3, and 6 h with AIP56. Cells without stimuli were used as control. The assay kit (Abcam) quantifies the cleavage of a substrate by caspase 3 or related caspases. The fluorescence (Ex/Em= 400/505 nm) was read and the fold-increase in caspase 3 activity was determined by comparing with the level determined in unstimulated cells cultured with L-15.

### Gene Expression

Total RNA was extracted from cells collected for innate immune response and apoptosis as following. After supernatant collection, wells were washed with HBSS and 50 µl of NZYol (NZYTech) reagent was added to each well, RNA was isolated following the manufacturer’s indications (NZY Total RNA Isolation kit) and resuspended in free nuclease water (NZYTech). RNA was quantified using the DS-11 Spectrophotometer (DeNovix) and samples were treated with DNase using RQ1 RNase-free DNase kit (Promega) following the manufacturer’s indications. First-strand cDNA was synthesized with NZY First-Strand cDNA Synthesis Kit (NZYTech). Quantitative PCR assays and primer design (**Table 1**) were performed as described by Machado et al. (24). The expression of the target gene was normalized using the average expression of European seabass *elongation factor 1α* (ef1α) and the *40s ribosomal protein* (40s). The target genes were selected according to the goal of each trial, direct immune mechanisms or apoptosis signaling. PCR efficiency and relative expression ratio of target gene in experimental groups versus those in control groups were calculated according Pfaffl method (50).

**Table 1:** Forward and reverse primers for real-time PCR.

Acronym	Gene	Gene Bank ID	Eff^1^	AT^2^	Product lenght^3^	Forward primer sequence (5’-3’)	Reverse primer sequence (5´-3´)	Trial
*ef1α*	Elongation factor 1α	AJ866727.1	96.45	57	144	AACTTCAACGCCCAGGTCAT	CTTCTTGCCAGAACGACGGT	Housekeeping
*40s*	40S ribosomal protein	HE978789.1	93	55	79	TGATTGTGACAGACCCTCGTG	CACAGAGCAATGGTGGGGAT	Housekeeping
*il1β*	Interleukin 1 β	AJ311925	96.70	57	105	AGCGACATGGTGCGATTTCT	CTCCTCTGCTGTGCTGATGT	Both trials
*mtor*	Mechanistic target of rapamycin	DLAgn_00134190	127.25	55	848	CAGAACCAAGGACGTGACGA	TGGTAGTAGAGGTCCCAGGC	Both trials
*il8*	Interleukin 8	AM490063.1	102.87	55	140	CGCTGCATCCAAACAGAGAGCAAAC	TCGGGGTCCAGGCAAACCTCTT	Both trials
*tnfα*	Tumor necrosis factor α	DQ070246.1	108.81	55	112	AGCCACAGGATCTGGAGCTA	GTCCGCTTCTGTAGCTGTCC	Both trials
*amd1*	Adenosylmethionine Decarboxylase 1	KM225770	118.64	57	63	CTGACGGAACTTACTGGACCATC	CGAAGCTGACGTAGGAGAACTC	Both trials
*sms*	Spermine synthase	DLAgn_00042290	111.71	55	132	GCACCTTTGGTTTCTCCTGA	AACTCAGTCCCACAGGGTTG	Both trials
*dnmt1*	DNA methyltransferase 1	DLAgn_00191600	84.37	60	193	ATGGCTTCACAAATGGCTCT	GATGGCTGTTTCCCACTGTT	Both trials
*dnmt3a*	DNA methyltransferase 3a	DLAgn_00025050	79.08	60	126	AAGTGGAAGATGGAGGCAGA	AGGCGATGGGTGTTTGATTA	Both trials
*dnmt3b*	DNA methyltransferase 3b	DLAgn_00125770	79.08	60	172	AAGCCCAAAGAAGGAGAGGA	GCAGGTTTCCCCAGAAGTATC	Both trials
ido2	Indoleamine -dioxygenase 2	DLAgn_00014730	108.20	55	74	TGAAGGTGTGAGCAATGAGC	CAAAGCACTGAATGGCTGAA	Both trials
*afmid*	Arylformamidase-like	DLAgn_00177950	128.26	55	112	CGTTTCCACCTGTTTGACCT	CCTAGCCTGCTGAAGGACTG	Both trials
*il6*	Interleukin 6	AM490062.1	134.62	55	81	AGGCACAGAGAACACGTCAAA	AAAAGGGTCAGGGCTGTCG	Immune
*il10*	Interleukin 10	AM268529.1	116.00	55	164	ACCCCGTTCGCTTGCCA	CATCTGGTGACATCACTC	Immune
*il13r*	Interleukin 13 receptor	KT809426.1	100.6183	55	118	AGGAACCGATGGAGTGAGTG	CCATAGCCATACCGCTTCAT	Immune
*cox2*	Cyclooxygenase 2	AJ630649.1	81.30	61	160	CATTCTTTGCCCAGCACTTCACC	AGCTTGCCATCCTTGAAGAGTC	Immune
*infγ*	Interferon γ	FQ310507.3	118.3801	55	194	GTACAGACAGGCGTCCAAAGCATCA	CAAACAGGGCAGCCGTCTCATCAA	Immune
*odc*	Ornithine decarboxylase	KM225771	111.71	60	69	GGGCTGTAGTTATGACACTGGCATCC	GCTGAATCTCCATCTTGCTTGCACAGT	Immune
*arg2*	Arginase 2	KM225768.1	90.25	57	145	TTGGCGACCTCAACTTCCAC	CCCAGCATGACAAGGGTGTG	Immune
*sod*	Superoxide dismutase	CX660893.1	103.0254	55	71	GGAGAGTGATTCAGCCCCTG	GGAAACCATGCTCACCAGGA	Immune
*nf-κb*	Nuclear Factor Kappa B	DLAgn_00239840	113.28	55	136	GCTGCGAGAAGAGAGGAAGA	GGTGAACTTTAACCGGACGA	Apoptosis
*p65*	Nuclear factor NF-kappa-B p65 subunit	DLAgn_00141590	97.63	62	204	GTGTGGTTTGTGTTGCCTTG	CCCTGAACCCATCTCGACTA	Apoptosis
*casp3*	Caspase 3	DQ345773.1	130.10	55	235	CTGATTTGGATCCAGGCATT	CGGTCGTAGTGTTCCTCCAT	Apoptosis
*casp8*	Caspase 8	DLAgn_00001990	107.71	60	140	CCGATGTTCTGGTAGCCATT	GAGGATGGTGGTCATGTCGT	Apoptosis
*casp9*	Caspase 9	DLAgn_00133660	103.73	60	127	TCTTGAGGAAAATGCGGTTA	TTTGCGGAGGAAGTTAAAGG	Apoptosis
*stat3*	Signal transducer/activator of transcription 3	DLAgn_00192560	110.68	55	275	GACATCAGCGGAAAGACCCA	GGGGTGACGCAGATGAACTT	Apoptosis

^1^The efficiency of PCR reactions was calculated from serial dilutions of tissue RT reactions in the validation procedure.

^2^Annealing temperature (°C).

^3^Amplicon (bp).

### Statistical Analysis

All results are expressed as mean ± standard deviation (mean ± SD). Data were analyzed for normality (Shapiro-Wilk’s W test) and homogeneity of variance (Levene’s test) and, when necessary, transformed before being treated statistically. Data were analyzed by one-way (MTT assay) or multifactorial ANOVA, with AA, time, and stimulus as factors, and followed by Tukey post-hoc test to identify differences between the experimental treatments. All statistical analyses were performed using the computer package STATISTICA 12 for WINDOWS. The level of significance used was p ≤ 0.05 for all statistical tests.

## Results

### MTT Assay

**Figure 2:**
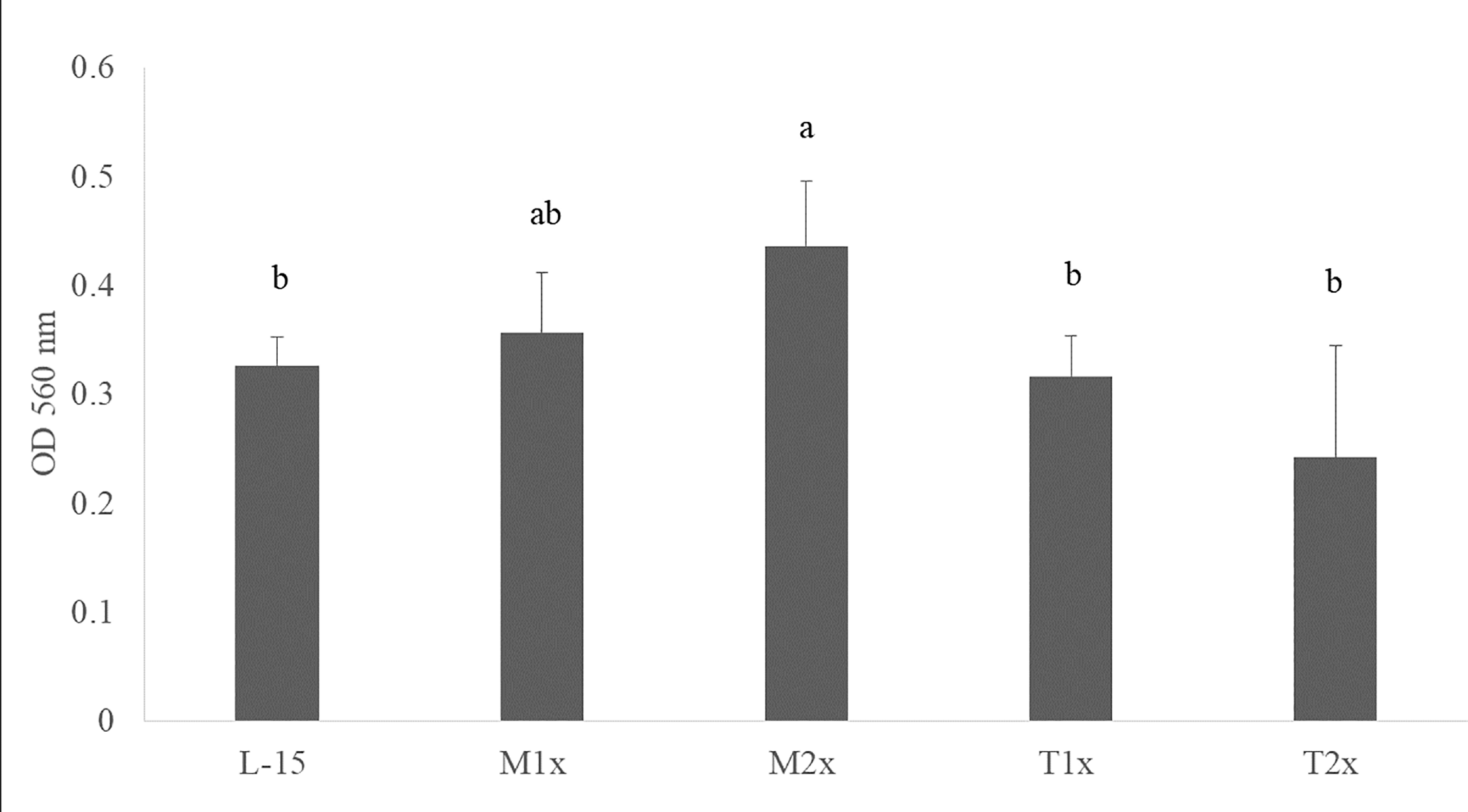
Cell viability after 24 h incubation with the different AA treatments. Values are presented as means ± SD (n = 12). P-values from one-way ANOVA (*p-value* = 0.001). Tukey *post hoc* test was used to identify differences in the experimental treatments. Different letters indicate differences among AA treatments.

After 24 h incubation of HKL with the different AA concentrations, the cell viability of the leucocytes cultured with M2x was increased compared to the control (L-15) as indicated by the different lower case letters in **Figure 2**. Tryptophan treatments (T1x and T2x) failed to alter HKL viability compared do L-15.

### Immune Response

#### ATP Production

**Figure 3:**
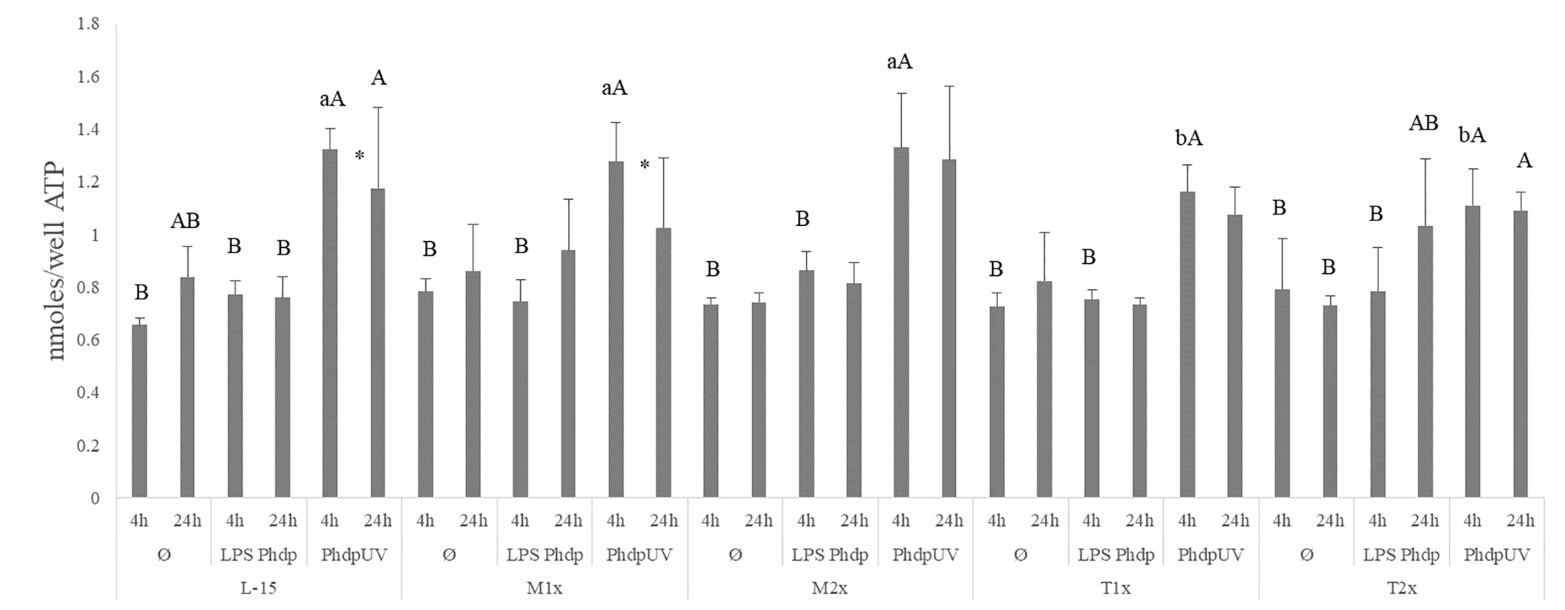
Extracellular ATP concentration in the supernatant of HKL subjected to the experimental treatments. Values are presented as means ± SD (n = 6). P-values from multifactorial ANOVA (*p-value* < 0.001). Tukey *post hoc* test was used to identify differences between the experimental treatments. Different low case letters indicate differences among AA treatments at the same time and stimuli, while capital letters denote statistically significant differences between stimuli, within the same AA treatment, at the same time. An asterisk indicates differences between times with the same stimulus.

All AA treatments presented higher ATP concentrations in response to *Phdp*UV compared to Ø and LPS*Phdp* after 4 h while at 24 h of incubation, only L-15 cells displayed higher ATP when incubated with *Phdp*UV than those incubated with LPS*Phdp* (capital letters in **Figure 3**). Methionine-treated cells (M1x and M2x) failed to alter ATP production compared to the control medium (L-15). Nevertheless, and as previously described ATP concentration was significantly increased in both methionine doses (M1x and M2x) in response to *Phdp*UV compared to the remaining stimulus at 4 h, with this production significantly decreasing at 24 h for the M1x. Similarly to methionine treatments, both T1x and T2x failed to increase ATP production in response to LPS*Phdp* compared to Ø whereas an increase of its production in response to *Phdp*UV at 4 h was observed. In fact, the higher tryptophan dose (T2x) showed the same pattern (increase of ATP production in response to *Phdp*UV compared to Ø) after 24 h of incubation. Finally, as pointed by the different lower case letters, T1x and T2x incubated for 4 h with *Phdp*UV presented a lower ATP concentration than cell culture in L-15 at the same time and stimuli.

#### TAC Concentration

**Figure 4:**
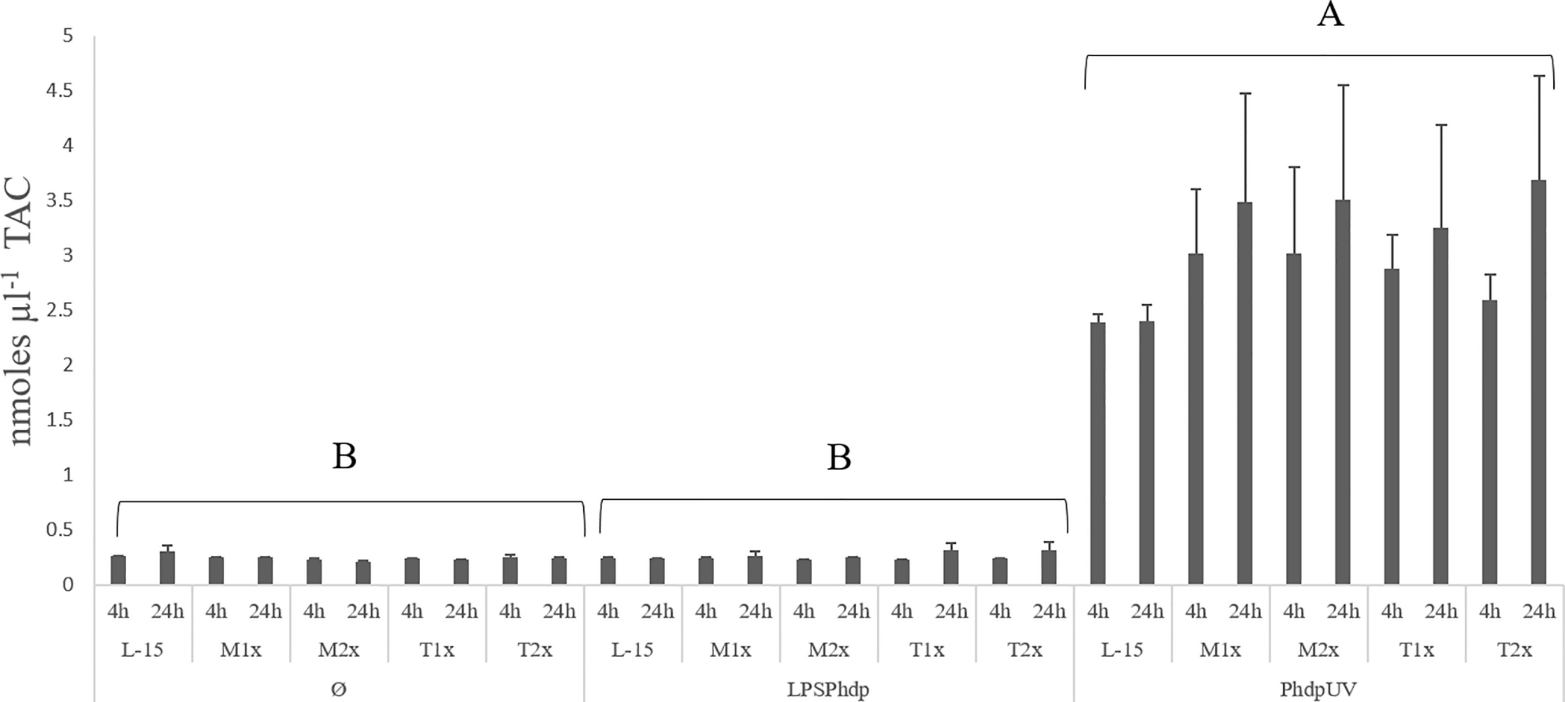
Extracellular TAC (total antioxidants concentration) in the supernatant of HKL subjected to the experimental treatments. Values are presented as means ± SD (n = 6). P-values from multifactorial ANOVA (*p-value* < 0.001). Tukey *post hoc* test was used to identify differences in the experimental treatments. Capital letters denote statistically significant differences between stimuli, within the same AA treatment, at the same time.

Results on TAC, presented in the **Figure 4**, were organized by stimulus contrary to the previous figure in order to properly point to the observed difference. A single significant increase in the total antioxidants was observed in the supernatant of HKL subjected to *Phdp*UV, regardless of exposure time and AA treatment, compared to the remaining stimulus.

#### Polyamines Concentration

**Figure 5:**
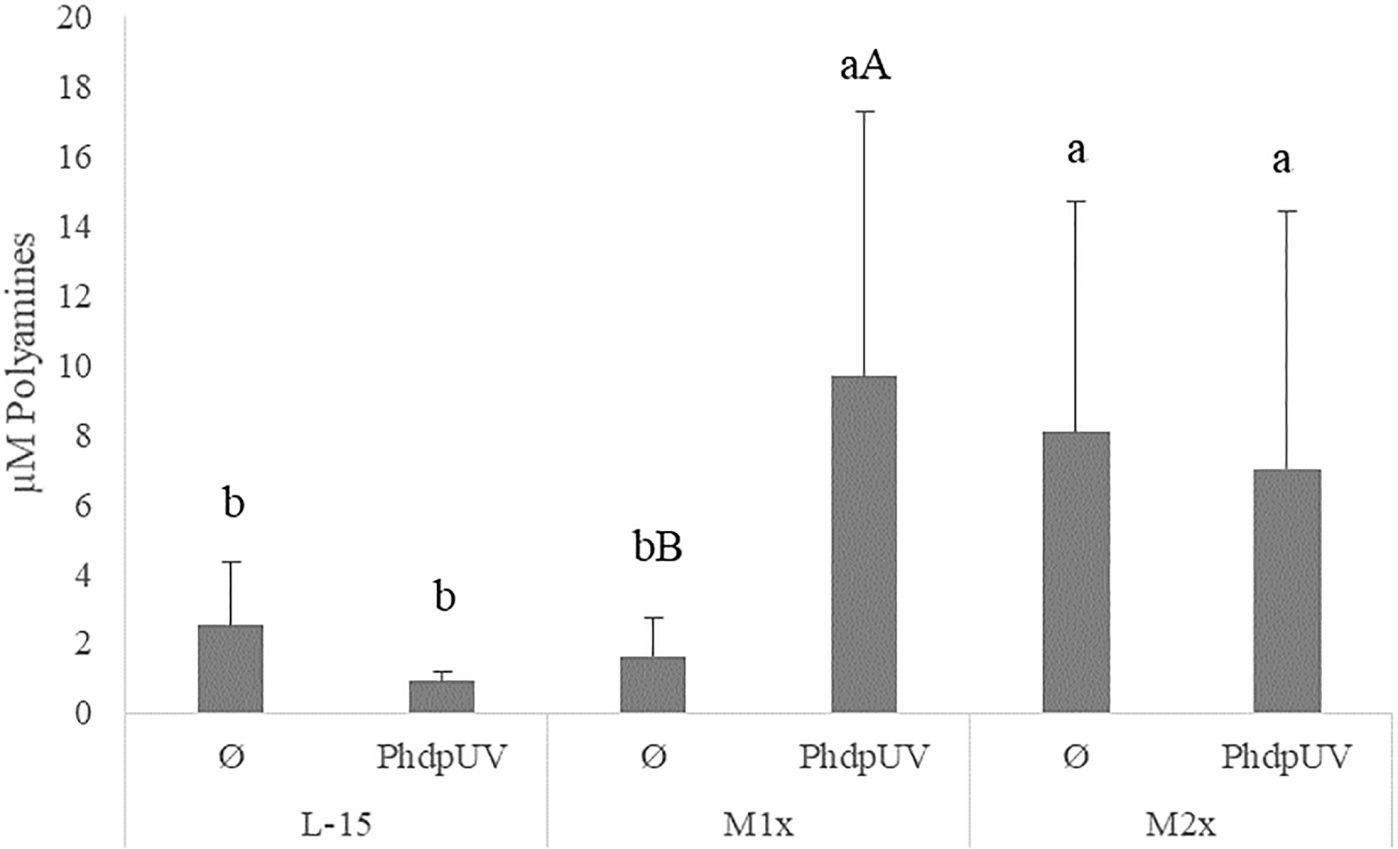
Extracellular polyamines concentration in the supernatant of HKL subjected to the experimental treatments. Values are presented as means ± SD (n = 6). P-values from multifactorial ANOVA (*p-value* = 0.048). Tukey *post hoc* test was used to identify differences in the experimental treatments. Different low case letters indicate differences among AA treatments at the same stimuli, while capital letters denote statistically significant differences between stimuli within the same AA treatment.

Since methionine has a recognized role in the polyamine biosynthesis pathway and no direct effect of tryptophan is expected, the assay was only performed in cells incubated with L-15, M1x, and M2x after 48 h stimulation with *Phdp*UV or unstimulated (Ø) (**Figure 5**). In the absence of an immune stimulus (Ø) cells cultured with M2x showed the ability to increased polyamine production compared to L-15, while M1x failed to do so (lower case letters). Besides that, upon immune stimulation by *Phdp*UV incubation for 48 h, both methionine doses, M1x and M2x, showed a higher polyamine production than those incubated in the standard medium, L-15.

#### NO Production

**Figure 6:**
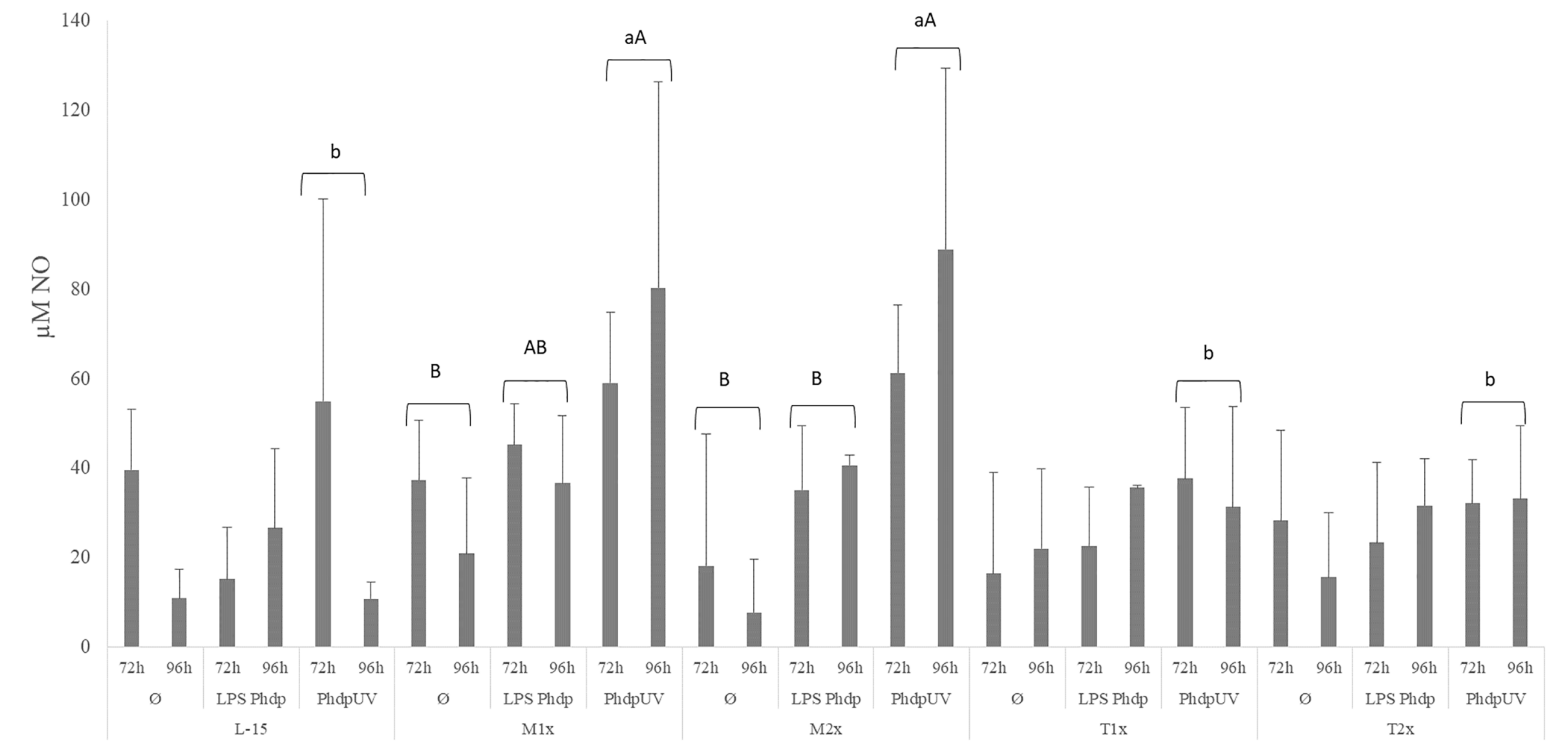
Extracellular nitric oxide concentration in the supernatant of HKL subjected to the experimental treatments. Values are presented as means ± SD (n = 6). P-values from multifactorial ANOVA (*p-value* = 0.009). Tukey *post hoc* test was used to identify differences in the experimental treatments. Different low case letters indicate differences among AA treatments while capital letters denote differences between stimuli.

Nitric oxide production is presented in **Figure 6**. The NO production in response to the different stimuli was modulated in both methionine doses. Both methionine treatments, M1x and M2x, presented higher NO production in response to *Phdp*UV than L-15 treatment, regardless of incubation time, while tryptophan failed to show any alterations compared to L-15 (lower case letters). In fact, in response to *Phdp*UV and regardless time of exposure, M1x enhanced NO concentration compared to Ø, while M2x-treated cells and stimulated with *Phdp*UV showed increased NO production compared to both Ø and LPS*Phdp* (capital letters).

#### Gene Expression

Gene expression results are presented in **Supplementary Table 1**. In response to stimuli, the mRNA transcripts of *il1β* and *dnmt1* were up-regulated in HKL exposed to *Phdp*UV compared to the remaining stimulus, regardless of AA treatment and time

**Figure 7:**
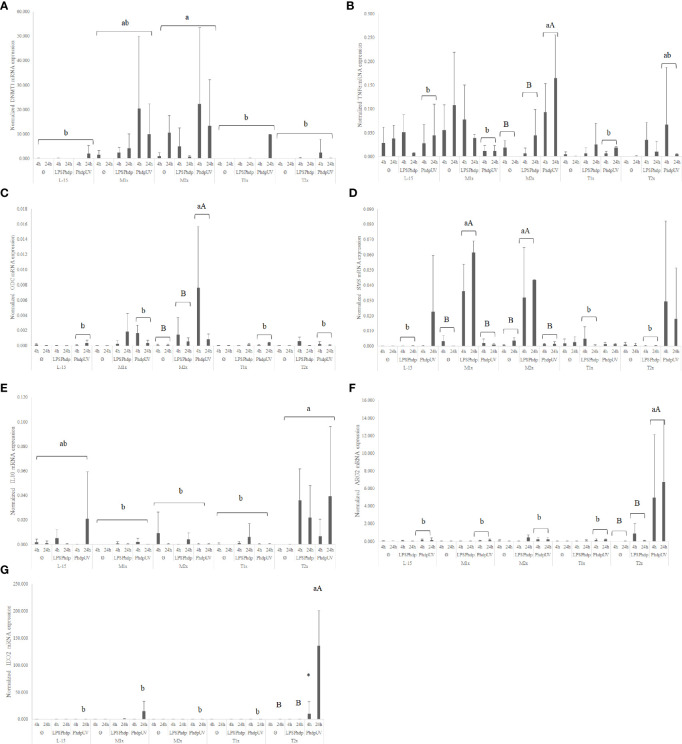
Quantitative expression of **(A)** DNA methyltransferase 1 (*p-value = 0.003*), **(B)** tumor necrosis factor α (*p-value = 0.001*), **(C)** ornithine decarboxylase (*p-value = 0.028*), **(D)** spermine synthase (*p-value <0.*001), **(E)** interleukin 10 (*p-value= 0.037*), **(F)** arginase 2 (*p-value < 0.001*) and **(G)** indoleamine dioxygenase 2 (*p-value < 0.001*) in HKL subjected to the experimental treatments. Values are presented as means ± SD (n = 6). Multifactorial ANOVA was followed by Tukey *post hoc* test was used to identify differences in the experimental treatments. Different low case letters indicate differences among AA treatments while capital letters denote statistically significant differences between stimuli. An asterisk indicates differences between times.

When looking to the methionine treatments, the highest medium supplementation (M2x) led to an increase of the overall expression of *dnmt1* when compared to the reaming AA treatments and disregarding stimuli or time of exposure (**Figure 7A**). Also, in response to *Phdp*UV, M2x treated-cells presented a significant up-regulation of *tnfα* (**Figure 7B**) and *odc* (**Figure 7C**) compared to both L-15 and M1x (lower case letters). In fact, this cells (M2x-cultured cells exposed to *Phdp*UV, regardless time) presented a significantly higher expression of *tnfα* and *odc* compared to LPS*Phdp* or Ø, respectively (capital letters). Methionine surplus (M1x and M2x) also increased *sms* (**Figure 7D**) mRNA expression when stimulated with LPS*Phdp*, compared to L-15. Also, the same treatments (M1x and M2x LPS*Phdp*) presented an improved expression compared to both unstimulated (Ø) and *Phdp*UV (capital letters).

Regarding tryptophan medium supplementation, HKL showed an up-regulation of the anti-inflammatory gene *il10* (**Figure 7E**) with the higher tested dose (T2x) presenting increased expression compared to M1x and M2x. However, as perceived by the different capital letters in **Figure 7F**, the highest tryptophan dose, T2x, up-regulated *arg2* transcripts in response to *Phdp*UV compared to the remaining AA treatments, and its unstimulated and LPS*Phdp* counterparts (capital letters). Additionally, T2x-treated cells showed an increase in time of *ido2* (**Figure 7G**) expression in response to *Phdp*UV with a significantly higher expression compared to all the remaining AA treatments (lower case letters) and its equivalents stimulated with LPS*Phdp* and Ø after a 24 h incubation (capital letters).

### Apoptotic Response

#### Amino Acids on AIP56 Cleaving Activity of the p65 Subunit

**Figure 8:**
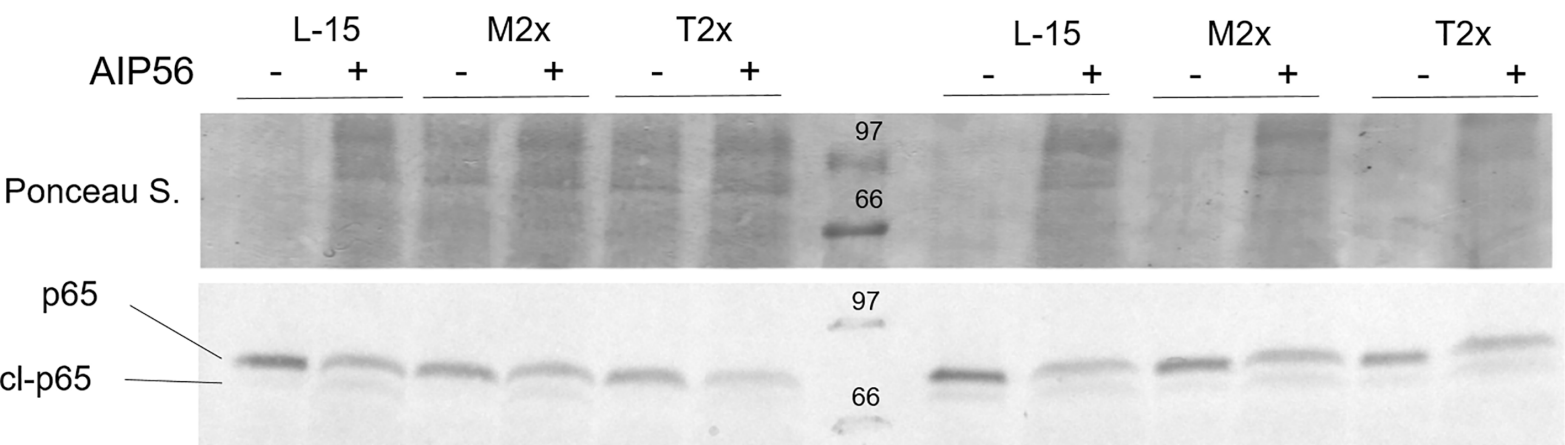
Example of a western blot of p65 cleavage in HKL lysates subjected to the experimental treatments and incubated for 2 h with Ø (-) or 2 µg ml^-1^ of AIP56 (+).

A total of four fish were used to test the activity of the AIP56 in the presence of the highest AA treatments by the evaluation of the p65 cleavage, as presented in **Figure 8**. Similarly to L-15, both M2x and T2x did not hampered AIP56 activity on p65 of HKL as seen by the lower detection in leucocytes incubated with the toxin. **Figure 8** presents the results of two biological replicate.

#### Caspase-3 Active

**Figure 9:**
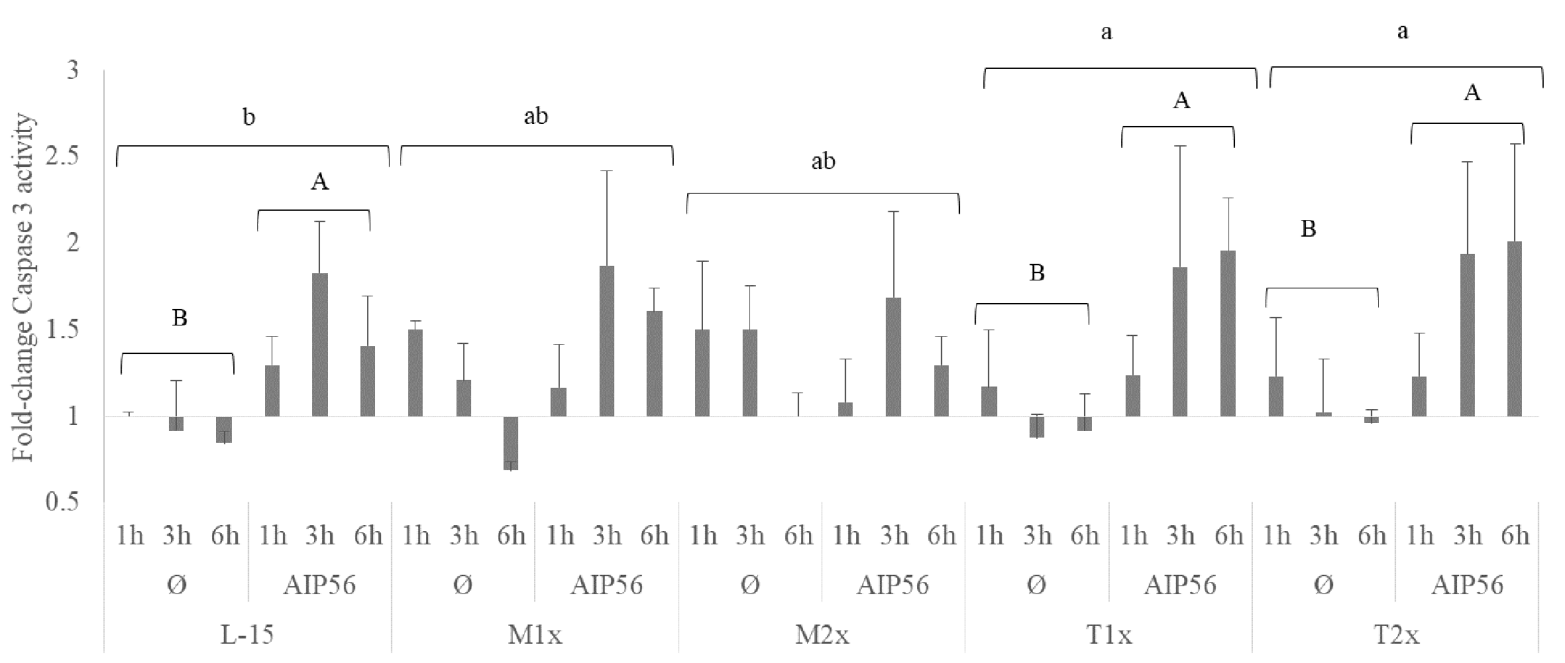
Caspase 3 activity of HKL subjected to the experimental treatments. Values are presented as means ± SD (n = 6). P-values from multifactorial ANOVA (*p- value* < 0.001). Tukey *post hoc* test was used to identify differences in the experimental treatments. Different low case letters indicate differences among AA treatments while capital letters denote statistically significant differences between stimuli for the same AA treatment.

A fold-change of caspase 3 activity relative to the unstimulated cells cultured with L-15 at 1 h was performed and presented in **Figure 9**. The activity of caspase 3 was evaluated in cells cultured with the different AA concentrations after 1, 3, and 6 h of stimulation with AIP56 or in the absence of the stimulus. No statistical modulation of caspase 3-activity was observed by methionine supplementation compared to L-15 and, despite the clear tendency, the expected increase of caspase 3- activity in response to AIP56 was not observed when compared to Ø, which could be explained by the relative high activity in methionine unstimulated (Ø) treatments (**Figure 8**). In the case of tryptophan, higher activity of caspase 3 was observed in HKL incubated with both supplementation levels, T1x and T2x, relative to L-15 and regardless of time or stimulus (lower case letters). Moreover, indicated by the capital letters, cells cultured in L-15, T1x, and T2x presented an increase of caspase 3- activity in response to AIP56.

#### Gene Expression

Due to the amount of data resulted from the gene expression, all data regarding is presented in **Supplementary table 2** including the main effects of the tested factors and the possible interactions.

**Figure 10:**
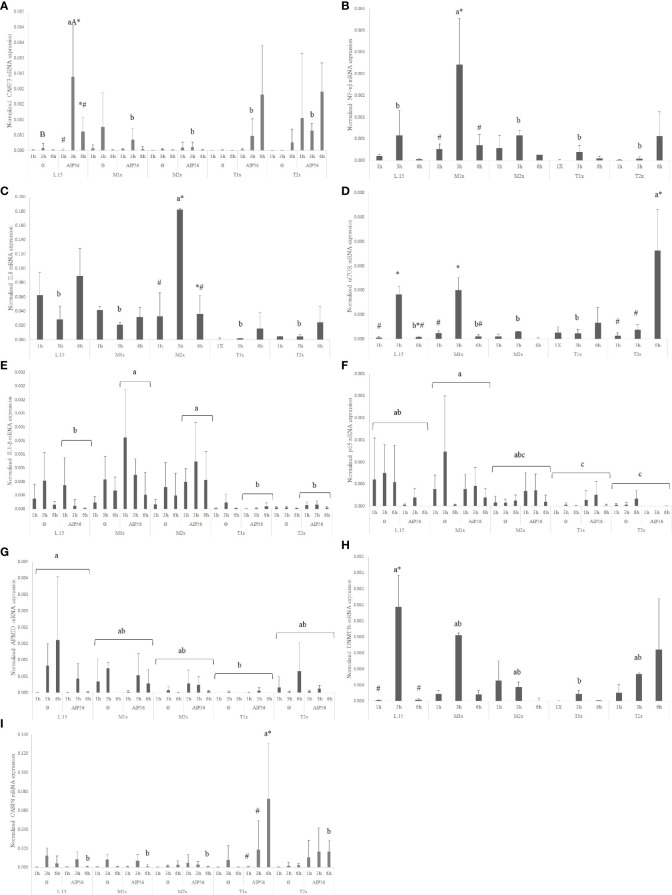
Quantitative expression of **(A)** caspase 3 (*p-value = 0.011*), **(B)** nuclear factor kappa B (*p-value = 0.012*), **(C)** interleukin 8 (*p-value 0.*0*15*), **(D)** mechanistic target of rapamycin (*p-value < 0.001*), **(E)** interleukin 1 β (*p-value = 0.032*), **(F)** transcription factor p65 (*p-value < 0.011*), **(G)** arylformamidase (*p-value < 0.025*), **(H)** DNA (cytosine-5-)-methyltransferase 3 beta (*p-value < 0.026*) and **(I)** caspase 8 (*p-value < 0.011*), in HKL subjected to the experimental treatments. Values are presented as means ± SD (n = 6). Multifactorial ANOVA was followed by Tukey *post hoc* test was used to identify differences in the experimental treatments. Different low case letters indicate differences among AA treatments while capital letters denote statistically significant differences between stimuli. Symbols indicate differences between times.

In response AIP56 stimuli, was observed a down-regulation of the *nf-κβ* regardless of AA treatment and time, while increasing the expression of *il1β* at 1h of exposure, regardless of AA (**Supplementary Table S2**). Additionally, L-15- incubated cells showed increased *casp3* expression at 3 h in response to AIP56 (**Figure 10A**), compared to Ø at the same time. L-15 cells showed increased *mto*r, *amd1*, and *dnmt3b* expression at 3 h compared to 1 and 6 h (**Supplementary Table S2**).

A general increase in the expression of pro-inflammatory genes, as *il1β*, *nf-κb*, and *il8* was found in response to methionine medium supplementation. Irrespective of stimuli, the expression of the pro-inflammatory signals *nf-κb* (**Figure 10B**) and *il8* (**Figure 10C**) *was* higher in M1x and M2x after 3 h, respectively, compared to L-15 and with both genes presenting a peak of expression at 3 h. M2x also showed higher *amd1* at 3 h, regardless of stimuli, and *mtor* was also found higher in M1x-treated cells at 3 h (**Figure 10D**). In response to AIP56, both methionine supplementation levels (M1x and M2x) allowed HKL to increase *il1β* (**Figure 10E**) transcripts compared to L-15 while decreasing the expression of *casp3* at 3 h (**Figure 10A**).

HKL incubated in a rich-tryptophan medium showed modulation of key apoptotic, catabolism, and nutrient sensing-related genes. Regardless of stimuli or incubation time, the expression of *p65* (**Figure 10F**) and *afmid* (**Figure 10G**) were down-regulated in both tryptophan levels and T1x, respectively, relative to L-15, while *dnmt3b* (**Figure 10H**) expression was down-regulated specifically at 3 h. Also, unstimulated (Ø) HKL cultured with T2x showed lower *sms* mRNA expression than HKL cultured with T1x and L-15 (**Supplementary Table S2**). Moreover, T2x showed decreased *amd1* expression at 3 h and increased *mto*r expression at 6 h, which presented a peak of expression at that time (**Supplementary Table S2**). In response to AIP56, *casp3* (**Figure 10A**) *transcripts* were down-regulated at 3 h in T1x and T2x compared to L-15, while *casp8* (**Figure 10I**), was found increased in T1x at 6 h incubation with AIP56 compared to all treatments.

## Discussion

The potential of methionine and tryptophan supplementation in the response of HKL upon immune stimulation with LPS-extracted and UV-inactivated *Phdp* was studied *in vitro*. Moreover, the HKL apoptosis instigated by AIP56 toxin was evaluated. The following discussion was assembled in order to individually debate each AA effect on the direct immune mechanism in response to the stimulus, on the AA-related pathways with close association with the innate mechanisms, and apoptotic signals.

The present study showed that, regardless of AA treatment, cell immune responses were not triggered following exposure to LPS*Phdp*. Most changes were observed in response to *Phdp*UV challenge, both at functional and transcriptional levels. Indeed, cultured cells exposed to LPS*Phdp* exhibited only one difference compared to the unstimulated cells in an AA surplus scenario (discussed later) while being generally surpassed by the *Phdp*UV effect. In response to *Phdp*UV, HKL increased *il1β* and *dnmt1* expressions, regardless AA treatment. *IL1β* is the key mediator of the inflammatory response produced by activated macrophages (51) and *DNMT1* is an enzyme that catalyzes the transfer of methyl groups derived from SAM to specific CpG structures of the DNA regulating gene expression (18). Together with *il1β* and *dnmt1* mRNA expressions, an increase of extracellular ATP and antioxidant concentrations (TAC) was observed in the HKL cultured in standard L-15 in response to *Phdp*UV comparatively to LPS*Phdp*. Increasing levels of extracellular ATP and antioxidant production are expected during inflammatory conditions (52, 53) since ATP acts as an inflammatory mediator during inflammation in macrophages (53). Also, antioxidants production are increased in response to inflammatory stimuli as a direct response to the enhanced production and release of reactive oxygen species (52). Likewise, the absence of pro-inflammatory indicators increment in response to LPS*Phdp* compared to the *Phdp*UV may point to other bacterial components rather than LPS that may be responsible for the observed response. Actually, the expected level of LPS within the *Phdp*UV inoculum at a concentration of 1 × 10^6^ CFU ml^-1^ was 0.006 µg ml^-1^, a value significantly lower than the 10 µg ml^-1^ LPS inoculum in LPS*Phdp*. Additionally, LPS recognition mechanisms by most teleost fish are still unknown, and there seems to be a lack of membrane receptor (i.e. Toll-like receptor) able to recognize LPS and induce inflammation (54). Indeed, Bi et al. (55) proposed the use of other strategies, such as outer membrane vesicles produced by Gram-negative bacteria, to serve as vehicle for LPS introduction to the intracellular host environment. These authors observed that, similarly to mammals, fish cytoplasmic NOD1 receptor is able to recognize LPS activating the NF- κB signal pathway and concomitant expression of pro-inflammatory signals. Hence, the limited effects observed in the present work upon purified LPS cell-stimulation could be explained by the lack of an intermediary vehicle to deliver LPS into the cell cytosol (56).

AIP56 is an exotoxin secreted by virulent *Phdp* and induces apoptotic macrophages death (57), impairing the host’s phagocytic capacity. Moreover, NF-κB, a key transcriptional factor in the initiation of inflammation (58), is the central target of cleavage by AIP56 (49). In the present work, the effectiveness of the toxin regardless AA treatment was confirmed by Western blot that displayed AIP56 ability to cleave the NF- κB- p65 subunit, together with a significant reduction of *nf-κb* expression.

### Methionine

In the present study, cultured cell viability was improved after 24 h incubation with a medium containing two times more methionine than the L-15 medium. The role of methionine in polyamine biosynthesis (19) has been proven to enhance European seabass leucocyte proliferation and response to infection *in vivo* (11, 14). Nonetheless, since proliferation and differentiation capacity of the present HKL cultured cells is very limited or even absent, increased viability strictly point to a potential improvement of cell fitness. A noteworthy finding is that both methionine supplementation levels led to an increase of polyamines cellular content after 48 h incubation period in response to *Phdp*UV. Moreover, the highest methionine supplementation level (i.e. M2x) presented increased polyamine content even in the absence of stimulus, together with the up-regulation of *dnmt1*, an enzyme that has a key role in the regulation of gene expression (18). In fact, recent *in vivo* study performed in European seabass juveniles (14) point to methionine surplus ability to increase the concentration of circulating leucocytes and neutrophils after 15 days of feeding. This supports the methionine-availability aptitude to modulate both aminopropylation (18) and methylation pathways (19) in the absence of immune stimuli.

Nevertheless, in response to an immune challenge, as LPS*Phdp*, both methionine supplementation levels improved *sms* mRNA expression. Spermine synthase, coded by *sms*, is an enzyme responsible for the conversion of polyamine spermidine into spermine. Moreover, when the immune insult was *Phdp*UV, HKL cultured in methionine-rich medium (M2x) were able to increase *odc* expression. Ornithine decarboxylase, coded by *odc*, is responsible for decarboxylation of ornithine to putrescine in the polyamine pathway also in macrophages (59). Also, an increase of tumor necrosis factor, *tnfα*, was observed. TNFα presents critical cell functions in cell proliferation, survival, differentiation, and apoptosis, with macrophages as major producers (60). It is then hypothesized that, together with an improvement of immune functions, modulation of methionine-related pathways, more precisely the aminopropylation and methylation routes, were stimulated. This agrees with previous *in vivo* studies that demonstrated an improvement of cellular immune status and immune response following an inflammatory insult, with modulation of the methionine-related polyamine biosynthesis pathway (11, 14). Moreover, both methionine-supplementation levels led to an improvement of NO production in response to inactivated bacteria (*Phdp*UV) compared to the control medium. Also, M1x and M2x were the only treatments that increased NO production in response to both unstimulated and LPS*Phdp* groups. As previously observed by Azeredo et al. (16) in response to UV-inactivated *Vibrio anguillarum*, the increased NO production in response to *Phdp*UV could be related to methionine ability to indirectly modulate respiratory burst mechanisms. As cysteine precursor, and consequently precursor of the free radical scavenger glutathione, methionine could have an important role in redox potential modulation (9).

When the apoptotic mechanisms were induced by the bacterial exotoxin AIP56 (57), methionine supplementation led to a down-regulation of *casp3* compared to the control after 3 h of stimulus, in spite of overall high caspase 3- activity (non-significant). Despite that, the decreased expression of *casp3* was accompanied by the higher expression of *nf-κb* in M1x-cultured cells at 3 h, regardless of stimulus. Caspase 3 is an apoptotic executioner caspase, triggered by the resulting chain reaction of the formerly activated initiator caspases (e.g. caspases 8 and 9) (61). Also, as previously discussed, NF-κB cleavage by AIP56 action (49) and may impair key inflammatory mechanisms such as the transcription of DNA and cytokine production (58), leucocyte recruitment, and cell survival (62). Knowing that *in vitro* methionine deprivation induces apoptosis (63, 64), it was considered that by the improvement of overall cell fitness, sustained by the increase of cell viability, polyamine content, NO production and increased expression of TNFα as well as methionine metabolism-related genes in response to and immune stimuli (LPS*Phdp* or *Phdp*UV), methionine supplementation may have contributed to the decrease of apoptotic signals, possibly alleviating AIP56-induced apoptosis signals. In fact, the expression of the cytokine IL1β, a pro-inflammatory cytokine induced by the NF-κB (39), was found increased in response to AIP56, showing signs of higher activity of the transcription factor.

Overall, present results point to an improvement of HKL immune response by methionine medium supplementation, mostly at the highest supplementation level (i.e. 1.5 mM). It is then proposed that the effects observed in recent *in vivo* works, where dietary methionine supplementation was able to enhance European seabass immune status and inflammatory machinery (11, 12, 65), rely on the amelioration of the pathways related to methionine catabolism, with a strict relationship with the leucocyte response, as the aminopropylation (18) and methylation pathways (19). Finally, the overall improved cell status in the methionine-supplemented medium seems to be in accordance with the protection against the apoptotic agent AIP56, and could be essential for the improvement of disease resistance against *Phdp*, as previously observed by Machado et al. (14).

### Tryptophan

Despite the tendency to reduce, the culture-medium supplementation of tryptophan during 24 h did not affect cell viability. Tryptophan involvement in the immune system relies mostly on the suppressor role of its metabolites, and despite a reduction of NO production was expected (16, 66), only a reduction of the extracellular ATP was detected in response to *Phdp*UV. The ATP released by the inflammatory cells acts as a pro-inflammatory autocrine/paracrine purinergic signal (67) and, by tryptophan supplementation, this indicator was reduced. Macrophages can be differentiated into distinct groups according to not only the inductor of differentiation but also to the inflammation stage and perceived signals (30, 35). Commonly baptized as SHIP [Sample, Heal (M2), Inhibit (M1), and Present (antigen)] (33), macrophages present some plasticity regarding their state, and type 2 macrophages (M2) are associated with the healing and repair phase of the inflammation. In the present study, HKL cultured in tryptophan enriched environment expressed more M2/repair type genotype, as suggested by the increased expression of arginase 2 (*arg2)* in response *Phdp*UV. Also, despite non-significant, a tendency to increase the expression of the anti-inflammatory cytokine interleukin 10 (*il10)* in response to LPS*Phdp* of T2x-treatmed cells compared to L-15 was observed. Polarized M2 macrophages are known to produce large quantities of *il10* (35, 68) and ornithine generated from arginase is associated with M2 phenotype (31). Despite contradictory, the specific function of the IDO enzyme is believed to be anti-inflammatory, as reviewed by Yang and Ming (69). Accordingly, in the present study, changes in the expression of *ido2* were also perceived. The indoleamine dioxygenase 2 enzyme mediates tryptophan catabolism by the kynurenine pathway in macrophages and relies on both tryptophan availability (5) and induction by an inflammatory stimulus (25). Thus, as expected, *ido2* was up-regulated in the higher tryptophan medium (T2x) after 24 h exposure to *Phdp*UV while LPS*Phdp* failed to induce expression.

In response to AIP56, cells cultured in tryptophan-supplemented medium (both T1x and T2x) presented an increase of caspase 3 activity relative to the control cells (L-15 medium cultured HKL). Curiously, the expression of *casp3*, an executioner caspase, was down-regulated relative to L-15 at 3 h of stimulus, while expression of *mtor*, which is involved in cell proliferation, was induced at 6 h in T2x-culture cells. It can be speculated that the apoptotic mechanisms were empowered by tryptophan surplus since, together with caspase 3 activity, the initiator caspase 8 expression increased at 6 h in cells cultured with T1x. The immunosuppressive role of tryptophan surplus is further supported by the observed down-regulation of *amd1* (19), *sms*, and *dntm3b* (18), which are genes with key roles in the support of cell proliferation and differentiation. Moreover, the expression of the target of AIP56 cleavage associated protein, the *p65* subunit, which is involved in the translocation and activation of NF-κB, was also down-regulated by tryptophan supplementation. Despite most differences presented in this study included both unstimulated and AIP56-stimulated cells, results seem to point to some level of aggravation of cell capacity to respond to AIP56 by tryptophan surplus.

Tryptophan supplementation above the level present in the L-15 medium, points to an attenuated inflammatory response, mostly sustained in the heal/repair extend of the immune system. Despite the lack of changes in the modulation of genes related to AA pathways, it is hypothesized that those mechanisms could have been prompted to sustain the M2/healing type of response to both LPS*Phdp* and *Phdp*UV. Some recent *in vivo* evidence indicates that the dietary supplementation of tryptophan in European seabass compromises leucocytes’ inflammatory response to UV-inactivated (11–13) and live *Phdp*, ultimately jeopardizing fish disease resistance to the pathogen (24). This is in agreement with the general increase of apoptotic indicators and compromised immune response to AIP56 in the cultured cells.

### An Integrated View of the Different Roles of Methionine and Tryptophan

The range of metabolic pathways that are dependent on a proper supply of specific AA unveils their importance in the support of metabolism and health (70). Immune homeostasis, which comprises host capacity to recognize, properly respond, and repair, relies on the adequate supply of specific AA. All these mechanisms are highly controlled and each pathway is initiated or inhibited according to numerous perceived signals. Nonetheless, specific AA can have a lead role in the pro-inflammatory part of the event (e.g. methionine) while others seem to play a more important role in the resolution of inflammation (e.g. tryptophan), presenting both the same importance in the overall immune response.

On the one hand, this was evident in the present study with methionine improving cell viability and polyamine production necessary for cell proliferation (19). These observations were accompanied by the increased expression of pro-inflammatory and methionine-related pathways indicators. With the increased expression of DNMT1 and up-regulation of *sms* and *odc*, the modulation of the methylation and aminopropylation pathways, respectively, are displayed. Finally, improved cell responses to an inflammatory agent were accompanied by lower signals of apoptosis by AIP56 with higher impression of pro-inflammatory indicators compared to the control treatment.

On the other hand, immune tolerance is of great importance, specifically in the limitation of self-damage and tryptophan seems to present a key role in such response. Most tryptophan-dependent pathways associated to the immune system are related to the assemblage of anti-inflammatory machinery. In macrophages, tryptophan consumption through the kynurenine pathway is catalyzed by IDO (71). In fact, IDO2 expression was found increased by tryptophan surplus and seemed to have induced signs of macrophages anti-inflammatory phenotype (i.e. high expression of arginase 2, *arg2*, in response to LPS*Phdp*) (68). Likewise, when submitted to AIP56 induced-apoptosis, cultured cells incubated with tryptophan presented increased signs of apoptosis and reduced expression of a critical regulator of immune and inflammatory responses (i.e. NF-κB P65), as well as proliferation and differentiation indicators (e.g. SMS, AMD1).

To conclude, in the present study it was specifically showed that methionine and tryptophan have their distinct roles in immune response, with different and contrasting outcomes by the modulation of individual key pathways. Methionine seems to positively contribute to the progress of inflammation, improving the underlying mechanisms activated in response to an inflammatory agent and lowering signals of apoptosis by AIP56, whereas tryptophan seems to presented a clear role in the tolerance process responsible for restriction of the pro-inflammatory cluster of the immune response. This is supported by the several signals of an attenuated inflammatory response to *Phdp*UV and lowered cell resilience to AIP56.

## Data Availability Statement

The datasets presented in this study can be found in online repositories. The names of the repository/repositories and accession number(s) can be found in the article/**Supplementary Material**.

## Ethics Statement

The animal study was reviewed and approved by the CIIMAR Animal Welfare Committee, carried out in a registered installation (N16091.UDER).

## Author Contributions

MM and BC conceived the experiments and MM conducted the experimental trials. CS purified the LPS*Phdp*. MM wrote the manuscript under the supervision of AO-T, CS, and BC. All authors contributed to the article and approved the submitted version.

## Funding

This work was partially supported by UIDB/04423/2020, UIDP/04423/2020 and INFLAMMAA (reference PTDC/CVT-CVT/32349/2017), financed by Portugal and the European Union through FEDER and COMPETE 2020, and national funds through Fundação para a Ciência e a Tecnologia (FCT, Portugal). MM and BC were supported by FCT, Portugal (SFRH/BD/108243/2015 and IF/00197/2015, respectively).

## Conflict of Interest

The authors declare that the research was conducted in the absence of any commercial or financial relationships that could be construed as a potential conflict of interest.
