# Supplementary material for: Methionine and Tryptophan Play Different Modulatory Roles in the European Seabass (Dicentrarchus labrax) Innate Immune Response and Apoptosis Signaling—An In Vitro Study
Source: Front Immunol. 2021 Mar 15;12:660448. doi: 10.3389/fimmu.2021.660448 (PMC8005646; doi:10.3389/fimmu.2021.660448)
Supplement: Supplementary file 1 [file DataSheet_1.docx]

Marina Machado^1,2,3,4*,^ Cláudia Serra^1^, Aires Oliva-Teles^1,5^ e Benjamín Costas^1,3^

^1^Centro Interdisciplinar de Investigação Marinha e Ambiental (CIIMAR), Terminal de Cruzeiros do Porto de Leixões, Av. General Norton de Matos s/n, 4450-208 Matosinhos
Portugal.

^2^ Instituto de Investigação e Inovação em Saúde (i3S), Universidade do Porto, Rua Alfredo Allen, 208, 4200-135 Porto, Portugal.

^3^ Instituto de Ciências Biomédicas Abel Salazar (ICBAS-UP), Universidade do Porto, Rua de Jorge Viterbo Ferreira nº 228, 4050-313 Porto, Portugal.

^4^ Instituto de Biologia Molecular e Celular, Universidade do Porto, Rua Alfredo Allen, 208, 4200-135 Porto, Portugal.

^5^ Departamento de Biologia, Faculdade de Ciências da Universidade do Porto (FCUP), Rua do Campo Alegre 1021/1055, 4169-007 Porto, Portugal.

***Corresponding authors:**

Marina Machado

[mcasimiro@ciimar.up.pt](mailto:mcasimiro@ciimar.up.pt)

Benjamín Costas

Bcostas@ciimar.up.pt

**Supplementary File**

**Table S1**. Quantitative expression of immune-related gene of head-kidney leucocytes subjected to the experimental conditions for 4 and 24 h.

| Parameters | | L-15 | | | | | | | | | | | | | | | | | | | | | | |
| --- | --- | --- | --- | --- | --- | --- | --- | --- | --- | --- | --- | --- | --- | --- | --- | --- | --- | --- | --- | --- | --- | --- | --- | --- |
|  |  | Ø | | | | | | |  | LPS*Phdp* | | | | | | |  | *Phdp*UV | | | | | | |
|  |  | 4h | | |  | 24h | | |  | 4h | | |  | 24h | | |  | 4h | | |  | 24h | | |
| *il1β* | Normalized mRNA expression | 0.02352 | ± | 0.02395 |  | 0.00045 | ± | 0.00047 |  | 0.04825 | ± | 0.02945 |  | 0.00969 | ± | 0.00000 |  | 0.46926 | ± | 0.70947 |  | 0.19036 | ± | 0.12150 |
| *il6* |  | 0.00002 | ± | 0.00000 |  | 0.00001 | ± | 0.00001 |  | 0.00001 | ± | 0.00002 |  | 0.00000 | ± | 0.00000 |  | 0.00027 | ± | 0.00026 |  | 0.00126 | ± | 0.00212 |
| *il10* |  | 0.00190 | ± | 0.00244 |  | 0.00116 | ± | 0.00183 |  | 0.00506 | ± | 0.00695 |  | 0.00039 | ± | 0.00019 |  | 0.00017 | ± | 0.00013 |  | 0.02096 | ± | 0.03819 |
| *il13r* |  | 0.05088 | ± | 0.06837 |  | 0.00499 | ± | 0.00723 |  | 0.02054 | ± | 0.03050 |  | 0.00823 | ± | 0.01135 |  | 0.02467 | ± | 0.00904 |  | 1.16113 | ± | 2.15455 |
| *tnfα* |  | 0.02915 | ± | 0.03325 |  | 0.03874 | ± | 0.02769 |  | 0.05098 | ± | 0.03670 |  | 0.00814 | ± | 0.00000 |  | 0.02836 | ± | 0.03904 |  | 0.04520 | ± | 0.06465 |
| *cox2* |  | 0.02101 | ± | 0.01757 |  | 0.00829 | ± | 0.00896 |  | 0.09419 | ± | 0.06746 |  | 0.01849 | ± | 0.01261 |  | 0.12261 | ± | 0.11966 |  | 14.80189 | ± | 25.43543 |
| *infγ* |  | 0.31876 | ± | 0.67963 |  | 0.00020 | ± | 0.00031 |  | 0.11751 | ± | 0.21252 |  | 0.00137 | ± | 0.00193 |  | 0.00054 | ± | 0.00081 |  | 0.31875 | ± | 0.62465 |
| *odc* |  | 0.00014 | ± | 0.00020 |  | 0.00003 | ± | 0.00004 |  | 0.00003 | ± | 0.00004 |  | 0.00001 | ± | 0.00001 |  | 0.00005 | ± | 0.00006 |  | 0.00038 | ± | 0.00037 |
| *arg2* |  | 0.04909 | ± | 0.03125 |  | 0.03938 | ± | 0.04419 |  | 0.05120 | ± | 0.05977 |  | 0.01745 | ± | 0.01150 |  | 0.13576 | ± | 0.14543 |  | 0.15259 | ± | 0.22946 |
| *mtor* |  | 0.00006 | ± | 0.00010 |  | 0.00000 | ± | 0.00000 |  | 0.00001 | ± | 0.00002 |  | 0.00002 | ± | 0.00002 |  | 0.00004 | ± | 0.00004 |  | 0.00238 | ± | 0.00434 |
| *sod* |  | 0.02487 | ± | 0.03820 |  | 0.06416 | ± | 0.10095 |  | 0.00373 | ± | 0.00536 |  | 0.00946 | ± | 0.00648 |  | 0.02551 | ± | 0.01716 |  | 0.58942 | ± | 1.03785 |
| *amd1* |  | 0.00116 | ± | 0.00168 |  | 0.00070 | ± | 0.00109 |  | 0.00032 | ± | 0.00047 |  | 0.00012 | ± | 0.00008 |  | 0.00140 | ± | 0.00141 |  | 0.00942 | ± | 0.01421 |
| *sms* |  | 0.00006 | ± | 0.00008 |  | 0.00001 | ± | 0.00000 |  | 0.00007 | ± | 0.00010 |  | 0.00024 | ± | 0.00031 |  | 0.00041 | ± | 0.00034 |  | 0.02255 | ± | 0.03714 |
| *dnmt1* |  | 0.12047 | ± | 0.15249 |  | 0.04477 | ± | 0.05488 |  | 0.07998 | ± | 0.11856 |  | 0.03680 | ± | 0.02686 |  | 0.04008 | ± | 0.04764 |  | 2.00043 | ± | 3.43748 |
| *dnmt3α* |  | 0.01621 | ± | 0.01896 |  | 0.31266 | ± | 0.53868 |  | 0.00028 | ± | 0.00010 |  | 0.01161 | ± | 0.01080 |  | 0.00944 | ± | 0.01222 |  | 0.33726 | ± | 0.55682 |
| *dnmt3β* |  | 0.28185 | ± | 0.32037 |  | 0.02784 | ± | 0.02065 |  | 0.13402 | ± | 0.20257 |  | 0.03771 | ± | 0.02369 |  | 0.05734 | ± | 0.05678 |  | 0.08807 | ± | 0.05618 |
| *ido2* |  | 0.04053 | ± | 0.07205 |  | 0.00003 | ± | 0.00004 |  | 0.00988 | ± | 0.01843 |  | 0.00004 | ± | 0.00004 |  | 0.00008 | ± | 0.00011 |  | 0.25050 | ± | 0.34629^b^ |
| *afmid1* |  | 0.00019 | ± | 0.00022 |  | 0.00002 | ± | 0.00002 |  | 0.00009 | ± | 0.00015 |  | 3.02892 | ± | 2.95910 |  | 0.00011 | ± | 0.00010 |  | 0.01048 | ± | 0.01831 |

| Parameters | | M1x | | | | | | | | | | | | | | | | | | | | | | |
| --- | --- | --- | --- | --- | --- | --- | --- | --- | --- | --- | --- | --- | --- | --- | --- | --- | --- | --- | --- | --- | --- | --- | --- | --- |
|  |  | Ø | | | | | | |  | LPS*Phdp* | | | | | | |  | *Phdp*UV | | | | | | |
|  |  | 4h | | |  | 24h | | |  | 4h | | |  | 24h | | |  | 4h | | |  | 24h | | |
| *il1β* | Normalized mRNA expression | 0.01528 | ± | 0.02938 |  | 0.00059 | ± | 0.00017 |  | 0.00304 | ± | 0.00419 |  | 0.03989 | ± | 0.04972 |  | 0.10272 | ± | 0.12548 |  | 0.06288 | ± | 0.08831 |
| *il6* |  | 0.00001 | ± | 0.00001 |  | 0.00000 | ± | 0.00000 |  | 0.00081 | ± | 0.00162 |  | 0.00003 | ± | 0.00003 |  | 0.00006 | ± | 0.00007 |  | 0.00005 | ± | 0.00004 |
| *il10* |  | 0.00007 | ± | 0.00010 |  | 0.00000 | ± | 0.00000 |  | 0.00085 | ± | 0.00125 |  | 0.00046 | ± | 0.00056 |  | 0.00197 | ± | 0.00306 |  | 0.00035 | ± | 0.00014 |
| *il13r* |  | 0.00476 | ± | 0.00677 |  | 0.00055 | ± | 0.00043 |  | 0.00145 | ± | 0.00193 |  | 0.00947 | ± | 0.01028 |  | 0.04162 | ± | 0.06585 |  | 0.01722 | ± | 0.01495 |
| *tnfα* |  | 0.05633 | ± | 0.05207 |  | 0.10825 | ± | 0.11093 |  | 0.07740 | ± | 0.07279 |  | 0.03948 | ± | 0.00701 |  | 0.01200 | ± | 0.01169 |  | 0.01198 | ± | 0.01197 |
| *cox2* |  | 0.00602 | ± | 0.00570 |  | 0.00685 | ± | 0.00483 |  | 0.01149 | ± | 0.00880 |  | 0.01557 | ± | 0.00910 |  | 0.01791 | ± | 0.01115 |  | 0.10689 | ± | 0.11382 |
| *infγ* |  | 0.02931 | ± | 0.05517 |  | 0.00000 | ± | 0.00000 |  | 0.00115 | ± | 0.00226 |  | 0.00171 | ± | 0.00210 |  | 0.00537 | ± | 0.00903 |  | 0.00015 | ± | 0.00014 |
| *odc* |  | 0.00003 | ± | 0.00002 |  | 0.00001 | ± | 0.00000 |  | 0.00025 | ± | 0.00044 |  | 0.00187 | ± | 0.00237 |  | 0.00169 | ± | 0.00100 |  | 0.00038 | ± | 0.00037 |
| *arg2* |  | 0.01467 | ± | 0.01143 |  | 0.02603 | ± | 0.02401 |  | 0.01456 | ± | 0.01412 |  | 0.02054 | ± | 0.01090 |  | 0.06917 | ± | 0.08329 |  | 0.15343 | ± | 0.12627 |
| *mtor* |  | 0.01574 | ± | 0.03519 |  | 0.00000 | ± | 0.00000 |  | 0.00000 | ± | 0.00000 |  | 0.00000 | ± | 0.00000 |  | 0.00013 | ± | 0.00021 |  | 0.00005 | ± | 0.00005 |
| *sod* |  | 0.00487 | ± | 0.00574 |  | 0.00262 | ± | 0.00195 |  | 0.00938 | ± | 0.01646 |  | 0.00654 | ± | 0.00770 |  | 0.08608 | ± | 0.13084 |  | 0.05394 | ± | 0.05848 |
| *amd1* |  | 0.01943 | ± | 0.04333 |  | 0.00044 | ± | 0.00059 |  | 0.02736 | ± | 0.05460 |  | 0.00019 | ± | 0.00023 |  | 0.00348 | ± | 0.00574 |  | 0.00024 | ± | 0.00012 |
| *sms* |  | 0.00338 | ± | 0.00367 |  | 0.00005 | ± | 0.00000 |  | 0.03625 | ± | 0.01767 |  | 0.06145 | ± | 0.00762 |  | 0.00198 | ± | 0.00270 |  | 0.00113 | ± | 0.00083 |
| *dnmt1* |  | 1.51356 | ± | 1.85949 |  | 0.00313 | ± | 0.00202 |  | 2.55029 | ± | 2.07626 |  | 4.17583 | ± | 5.86319 |  | 20.36091 | ± | 29.60585 |  | 10.00945 | ± | 12.36575 |
| *dnmt3α* |  | 0.00192 | ± | 0.00143 |  | 0.00042 | ± | 0.00012 |  | 0.01177 | ± | 0.01179 |  | 0.27039 | ± | 0.25182 |  | 0.00420 | ± | 0.00488 |  | 0.11103 | ± | 0.16095 |
| *dnmt3β* |  | 0.01106 | ± | 0.00625 |  | 0.00967 | ± | 0.00107 |  | 0.01363 | ± | 0.01545 |  | 0.01481 | ± | 0.01654 |  | 0.05809 | ± | 0.07673 |  | 0.11742 | ± | 0.09345 |
| *ido2* |  | 0.00005 | ± | 0.00004 |  | 0.00019 | ± | 0.00025 |  | 0.00003 | ± | 0.00002 |  | 0.54969 | ± | 0.71399 |  | 0.02248 | ± | 0.03788 |  | 14.72889 | ± | 17.99144^b^ |
| *afmid1* |  | 2.32149 | ± | 5.15981 |  | 0.00000 | ± | 0.00000 |  | 0.03124 | ± | 0.06248 |  | 0.00001 | ± | 0.00001 |  | 0.00014 | ± | 0.00021 |  | 0.00011 | ± | 0.00013 |

| Parameters | | M2x | | | | | | | | | | | | | | | | | | | | | | |
| --- | --- | --- | --- | --- | --- | --- | --- | --- | --- | --- | --- | --- | --- | --- | --- | --- | --- | --- | --- | --- | --- | --- | --- | --- |
|  |  | Ø | | | | | | |  | LPS*Phdp* | | | | | | |  | *Phdp*UV | | | | | | |
|  |  | 4h | | |  | 24h | | |  | 4h | | |  | 24h | | |  | 4h | | |  | 24h | | |
| *il1β* | Normalized mRNA expression | 0.04163 | ± | 0.07832 |  | 0.00032 | ± | 0.00025 |  | 0.00052 | ± | 0.00050 |  | 0.00004 | ± | 0.00003 |  | 0.07226 | ± | 0.09837 |  | 0.08555 | ± | 0.11732 |
| *il6* |  | 0.00156 | ± | 0.00268 |  | 0.00004 | ± | 0.00003 |  | 0.00019 | ± | 0.00035 |  | 0.00011 | ± | 0.00012 |  | 0.00018 | ± | 0.00016 |  | 0.00010 | ± | 0.00009 |
| *il10* |  | 0.00913 | ± | 0.01749 |  | 0.00049 | ± | 0.00039 |  | 0.00014 | ± | 0.00026 |  | 0.00433 | ± | 0.00513 |  | 0.00050 | ± | 0.00053 |  | 0.00047 | ± | 0.00037 |
| *il13r* |  | 0.04060 | ± | 0.07370 |  | 0.00756 | ± | 0.00570 |  | 0.00255 | ± | 0.00304 |  | 0.22331 | ± | 0.19627 |  | 0.02940 | ± | 0.03004 |  | 0.02436 | ± | 0.01537 |
| *tnfα* |  | 0.01944 | ± | 0.01420 |  | 0.00005 | ± | 0.00005 |  | 0.00705 | ± | 0.01128 |  | 0.04509 | ± | 0.05360 |  | 0.09408 | ± | 0.05908 |  | 0.16519 | ± | 0.09044 |
| *cox2* |  | 0.13692 | ± | 0.17028 |  | 0.02151 | ± | 0.00605 |  | 0.84431 | ± | 1.04126 |  | 0.18707 | ± | 0.26524 |  | 0.13215 | ± | 0.12096 |  | 0.21888 | ± | 0.20818 |
| *infγ* |  | 0.00025 | ± | 0.00038 |  | 0.00105 | ± | 0.00104 |  | 0.00094 | ± | 0.00185 |  | 0.12360 | ± | 0.14778 |  | 0.00079 | ± | 0.00083 |  | 0.00154 | ± | 0.00267 |
| *odc* |  | 0.00008 | ± | 0.00010 |  | 0.00011 | ± | 0.00009 |  | 0.00146 | ± | 0.00224 |  | 0.00055 | ± | 0.00049 |  | 0.00763 | ± | 0.00802 |  | 0.00086 | ± | 0.00067 |
| *arg2* |  | 0.07906 | ± | 0.07649 |  | 0.03899 | ± | 0.02404 |  | 0.02528 | ± | 0.02108 |  | 0.45437 | ± | 0.30033 |  | 0.22920 | ± | 0.22435 |  | 0.22516 | ± | 0.19664 |
| *mtor* |  | 0.00000 | ± | 0.00000 |  | 0.00001 | ± | 0.00001 |  | 0.00000 | ± | 0.00000 |  | 0.00085 | ± | 0.00110 |  | 0.00006 | ± | 0.00006 |  | 0.00007 | ± | 0.00005 |
| *sod* |  | 0.08937 | ± | 0.10780 |  | 0.01727 | ± | 0.01502 |  | 0.01920 | ± | 0.03388 |  | 0.04094 | ± | 0.03294 |  | 0.02197 | ± | 0.01334 |  | 0.06069 | ± | 0.03249 |
| *amd1* |  | 0.00038 | ± | 0.00047 |  | 0.00025 | ± | 0.00014 |  | 0.00017 | ± | 0.00022 |  | 0.00731 | ± | 0.00690 |  | 0.00079 | ± | 0.00095 |  | 0.00127 | ± | 0.00120 |
| *sms* |  | 0.00044 | ± | 0.00050 |  | 0.00344 | ± | 0.00220 |  | 0.03194 | ± | 0.03268 |  | 0.04352 | ± | 0.00000 |  | 0.00118 | ± | 0.00078 |  | 0.00161 | ± | 0.00128 |
| *dnmt1* |  | 0.96321 | ± | 1.39749 |  | 10.58264 | ± | 7.08795 |  | 4.97386 | ± | 7.55288 |  | 0.70402 | ± | 0.43772 |  | 22.22704 | ± | 31.08880 |  | 13.32132 | ± | 18.86288 |
| *dnmt3α* |  | 0.01567 | ± | 0.01413 |  | 0.01118 | ± | 0.01323 |  | 0.00124 | ± | 0.00133 |  | 0.04459 | ± | 0.05441 |  | 0.43272 | ± | 0.65902 |  | 0.00405 | ± | 0.00272 |
| *dnmt3β* |  | 0.04884 | ± | 0.04783 |  | 0.05309 | ± | 0.01711 |  | 0.01789 | ± | 0.02290 |  | 0.70871 | ± | 0.81486 |  | 0.17500 | ± | 0.19905 |  | 0.15845 | ± | 0.19441 |
| *ido2* |  | 0.00060 | ± | 0.00104 |  | 0.00006 | ± | 0.00005 |  | 0.00048 | ± | 0.00078 |  | 0.00239 | ± | 0.00269 |  | 0.03592 | ± | 0.06480 |  | 0.25252 | ± | 0.36569^b^ |
| *afmid1* |  | 0.01882 | ± | 0.03684 |  | 0.00002 | ± | 0.00001 |  | 0.00001 | ± | 0.00001 |  | 0.00995 | ± | 0.00744 |  | 0.00015 | ± | 0.00019 |  | 0.00011 | ± | 0.00008 |

| Parameters | | T1x | | | | | | | | | | | | | | | | | | | | | | |
| --- | --- | --- | --- | --- | --- | --- | --- | --- | --- | --- | --- | --- | --- | --- | --- | --- | --- | --- | --- | --- | --- | --- | --- | --- |
|  |  | Ø | | | | | | |  | LPS*Phdp* | | | | | | |  | *Phdp*UV | | | | | | |
|  |  | 4h | | |  | 24h | | |  | 4h | | |  | 24h | | |  | 4h | | |  | 24h | | |
| *il1β* | Normalized mRNA expression | 0.00017 | ± | 0.00000 |  | 0.00008 | ± | 0.00003 |  | 0.00107 | ± | 0.00124 |  | 0.00095 | ± | 0.00105 |  | 0.09479 | ± | 0.13116 |  | 0.09312 | ± | 0.10648 |
| *il6* |  | 0.00009 | ± | 0.00015 |  | 0.00000 | ± | 0.00000 |  | 0.00029 | ± | 0.00057 |  | 0.00030 | ± | 0.00051 |  | 0.00015 | ± | 0.00013 |  | 0.00014 | ± | 0.00007 |
| *il10* |  | 0.00048 | ± | 0.00066 |  | 0.00004 | ± | 0.00004 |  | 0.00109 | ± | 0.00149 |  | 0.00617 | ± | 0.01074 |  | 0.00065 | ± | 0.00046 |  | 0.00033 | ± | 0.00028 |
| *il13r* |  | 0.01717 | ± | 0.03379 |  | 0.00203 | ± | 0.00180 |  | 0.01737 | ± | 0.01532 |  | 0.05262 | ± | 0.05295 |  | 0.03582 | ± | 0.03595 |  | 0.01454 | ± | 0.01345 |
| *tnfα* |  | 0.00477 | ± | 0.00509 |  | 0.00054 | ± | 0.00042 |  | 0.00744 | ± | 0.01115 |  | 0.02597 | ± | 0.04445 |  | 0.00742 | ± | 0.00438 |  | 0.01942 | ± | 0.00156 |
| *cox2* |  | 0.01523 | ± | 0.00860 |  | 0.00946 | ± | 0.00621 |  | 0.01082 | ± | 0.00315 |  | 0.05225 | ± | 0.05808 |  | 1.57348 | ± | 3.27992 |  | 0.08443 | ± | 0.03097 |
| *infγ* |  | 0.05099 | ± | 0.10942 |  | 0.12229 | ± | 0.21176 |  | 0.00143 | ± | 0.00164 |  | 0.01215 | ± | 0.02120 |  | 0.00124 | ± | 0.00150 |  | 0.00025 | ± | 0.00014 |
| *odc* |  | 0.00004 | ± | 0.00004 |  | 0.00004 | ± | 0.00003 |  | 0.00003 | ± | 0.00003 |  | 0.00017 | ± | 0.00018 |  | 0.00007 | ± | 0.00006 |  | 0.00039 | ± | 0.00002 |
| *arg2* |  | 0.03013 | ± | 0.02320 |  | 0.03425 | ± | 0.02528 |  | 0.03566 | ± | 0.03064 |  | 0.07623 | ± | 0.08686 |  | 0.16448 | ± | 0.16055 |  | 0.18089 | ± | 0.09058 |
| *mtor* |  | 0.00000 | ± | 0.00001 |  | 0.00000 | ± | 0.00000 |  | 0.00000 | ± | 0.00000 |  | 0.00003 | ± | 0.00003 |  | 0.00007 | ± | 0.00006 |  | 0.00004 | ± | 0.00001 |
| *sod* |  | 0.01788 | ± | 0.01302 |  | 0.01026 | ± | 0.01187 |  | 0.01362 | ± | 0.01777 |  | 0.03665 | ± | 0.03403 |  | 0.02675 | ± | 0.01599 |  | 0.03428 | ± | 0.02090 |
| *amd1* |  | 0.00042 | ± | 0.00050 |  | 0.00008 | ± | 0.00004 |  | 0.00034 | ± | 0.00033 |  | 0.00203 | ± | 0.00220 |  | 0.01643 | ± | 0.03422 |  | 0.00037 | ± | 0.00020 |
| *sms* |  | 0.00181 | ± | 0.00290 |  | 0.00244 | ± | 0.00373 |  | 0.00489 | ± | 0.00781 |  | 0.00038 | ± | 0.00045 |  | 0.00140 | ± | 0.00108 |  | 0.00140 | ± | 0.00053 |
| *dnmt1* |  | 0.01983 | ± | 0.02033 |  | 0.00760 | ± | 0.00796 |  | 0.02150 | ± | 0.01537 |  | 0.08603 | ± | 0.11790 |  | 0.05245 | ± | 0.04313 |  | 9.87772 | ± | 0.01050 |
| *dnmt3α* |  | 0.00382 | ± | 0.00745 |  | 0.00079 | ± | 0.00093 |  | 0.01598 | ± | 0.03219 |  | 0.00588 | ± | 0.00895 |  | 0.00511 | ± | 0.00598 |  | 0.00255 | ± | 0.00150 |
| *dnmt3β* |  | 0.08277 | ± | 0.14760 |  | 0.02407 | ± | 0.02564 |  | 0.27396 | ± | 0.50998 |  | 0.12639 | ± | 0.17193 |  | 0.16842 | ± | 0.16357 |  | 0.14882 | ± | 0.05215 |
| *ido2* |  | 0.00013 | ± | 0.00023 |  | 0.00002 | ± | 0.00001 |  | 0.00007 | ± | 0.00004 |  | 0.00030 | ± | 0.00046 |  | 0.14858 | ± | 0.15463 |  | 0.29297 | ± | 0.21041^b^ |
| *afmid1* |  | 0.05160 | ± | 0.09435 |  | 0.00001 | ± | 0.00000 |  | 0.00002 | ± | 0.00001 |  | 0.00014 | ± | 0.00019 |  | 0.00018 | ± | 0.00020 |  | 0.00011 | ± | 0.00009 |

| Parameters | | T2x | | | | | | | | | | | | | | | | | | | | | | |
| --- | --- | --- | --- | --- | --- | --- | --- | --- | --- | --- | --- | --- | --- | --- | --- | --- | --- | --- | --- | --- | --- | --- | --- | --- |
|  |  | Ø | | | | | | |  | LPS*Phdp* | | | | | | |  | *Phdp*UV | | | | | | |
|  |  | 4h | | |  | 24h | | |  | 4h | | |  | 24h | | |  | 4h | | |  | 24h | | |
| *il1β* | Normalized mRNA expression | 0.00032 | ± | 0.00011 |  | 0.00704 | ± | 0.01211 |  | 0.02231 | ± | 0.02862 |  | 0.00197 | ± | 0.00315 |  | 0.13293 | ± | 0.18460 |  | 0.09717 | ± | 0.14005 |
| *il6* |  | 0.00000 | ± | 0.00001 |  | 0.00001 | ± | 0.00000 |  | 0.00208 | ± | 0.00269 |  | 0.00008 | ± | 0.00010 |  | 0.00360 | ± | 0.00808 |  | 0.00162 | ± | 0.00175 |
| *il10* |  | 0.00001 | ± | 0.00000 |  | 0.00010 | ± | 0.00009 |  | 0.03603 | ± | 0.02582 |  | 0.02206 | ± | 0.02593 |  | 0.00679 | ± | 0.01393 |  | 0.03943 | ± | 0.05682 |
| *il13r* |  | 0.00132 | ± | 0.00053 |  | 0.00098 | ± | 0.00094 |  | 0.09802 | ± | 0.16158 |  | 0.02236 | ± | 0.03242 |  | 1.43554 | ± | 3.34185 |  | 0.02508 | ± | 0.01693 |
| *tnfα* |  | 0.00034 | ± | 0.00033 |  | 0.00147 | ± | 0.00129 |  | 0.03505 | ± | 0.03628 |  | 0.01010 | ± | 0.02210 |  | 0.06710 | ± | 0.12085 |  | 0.00515 | ± | 0.00082 |
| *cox2* |  | 0.00309 | ± | 0.00186 |  | 0.02018 | ± | 0.02426 |  | 0.06862 | ± | 0.07775 |  | 0.06741 | ± | 0.06862 |  | 7.12424 | ± | 16.30867 |  | 0.26114 | ± | 0.18613 |
| *infγ* |  | 0.00503 | ± | 0.01121 |  | 0.02092 | ± | 0.04183 |  | 0.03472 | ± | 0.05993 |  | 0.21764 | ± | 0.47881 |  | 0.00369 | ± | 0.00773 |  | 0.00057 | ± | 0.00039 |
| *odc* |  | 0.00001 | ± | 0.00001 |  | 0.00001 | ± | 0.00000 |  | 0.00060 | ± | 0.00053 |  | 0.00005 | ± | 0.00005 |  | 0.00020 | ± | 0.00031 |  | 0.00007 | ± | 0.00009 |
| *arg2* |  | 0.00441 | ± | 0.00150 |  | 0.03214 | ± | 0.03215 |  | 0.90571 | ± | 1.08797 |  | 0.06799 | ± | 0.04576 |  | 4.96801 | ± | 7.18105 |  | 6.74357 | ± | 7.06041 |
| *mtor* |  | 0.00000 | ± | 0.00000 |  | 0.00001 | ± | 0.00001 |  | 0.00005 | ± | 0.00006 |  | 0.00641 | ± | 0.01428 |  | 0.00275 | ± | 0.00619 |  | 0.00009 | ± | 0.00008 |
| *sod* |  | 0.00174 | ± | 0.00323 |  | 0.00460 | ± | 0.00345 |  | 0.10260 | ± | 0.09421 |  | 0.01213 | ± | 0.01271 |  | 0.06897 | ± | 0.11040 |  | 3.36486 | ± | 6.47526 |
| *amd1* |  | 0.00002 | ± | 0.00002 |  | 0.00010 | ± | 0.00007 |  | 0.00234 | ± | 0.00348 |  | 0.00062 | ± | 0.00060 |  | 1.08965 | ± | 2.57593 |  | 0.00098 | ± | 0.00100 |
| *sms* |  | 0.00107 | ± | 0.00141 |  | 0.00067 | ± | 0.00125 |  | 0.00037 | ± | 0.00030 |  | 0.00013 | ± | 0.00024 |  | 0.02949 | ± | 0.05279 |  | 0.01792 | ± | 0.03348 |
| *dnmt1* |  | 0.00334 | ± | 0.00309 |  | 0.00348 | ± | 0.00127 |  | 0.22323 | ± | 0.33043 |  | 0.03934 | ± | 0.03917 |  | 2.42893 | ± | 5.39299 |  | 0.10160 | ± | 0.11916 |
| *dnmt3α* |  | 0.00008 | ± | 0.00004 |  | 0.00013 | ± | 0.00004 |  | 0.01948 | ± | 0.03068 |  | 0.01110 | ± | 0.01536 |  | 0.24376 | ± | 0.54197 |  | 0.01620 | ± | 0.02235 |
| *dnmt3β* |  | 0.00361 | ± | 0.00148 |  | 0.00581 | ± | 0.00137 |  | 0.33508 | ± | 0.50213 |  | 0.18615 | ± | 0.27085 |  | 0.61752 | ± | 1.22444 |  | 0.38749 | ± | 0.33623 |
| *ido2* |  | 0.00001 | ± | 0.00001 |  | 0.00162 | ± | 0.00210^B^ |  | 0.00135 | ± | 0.00166 |  | 0.01562 | ± | 0.02340^B^ |  | 9.72314 | ± | 22.60860* |  | 135.97803 | ± | 64.20682^aA^ |
| *afmid1* |  | 0.00000 | ± | 0.00000 |  | 0.00608 | ± | 0.01052 |  | 0.00141 | ± | 0.00141 |  | 0.00044 | ± | 0.00086 |  | 0.00584 | ± | 0.01319 |  | 0.03193 | ± | 0.05310 |

| Multifactorial ANOVA | | |  |  |  |  |  |  |  |  |  |  |  |  |  |  |  |
| --- | --- | --- | --- | --- | --- | --- | --- | --- | --- | --- | --- | --- | --- | --- | --- | --- | --- |
|  | AA | Stimuli | Time | AA × Stimuli | AA × Time | Stimuli × Time | AA × Stimuli × Time |  | AA | | | | |  | Stimuli | | |
|  |  |  |  |  |  |  |  |  | L-15 | M1x | M2x | T1x | T2x |  | Ø | LPS*Phdp* | *Phdp*UV |
| *il1β* | - | <0.001 | - | - | - | - | - |  | - | - | - | - | - |  | B | B | A |
| *il6* | - | - | - | - | - | - | - |  | - | - | - | - | - |  | - | - | - |
| *il10* | <0.001 | - | - | - | - | - | - |  | ab | b | b | b | a |  | - | - | - |
| *il13r* | - | - | - | - | - | - | - |  | - | - | - | - | - |  | - | - | - |
| *tnfα* | 0.006 | - | - | 0.001 | - | - | - |  | abc | ab | a | c | b |  | - | - | - |
| *cox2* | - | - | - | - | - | - | - |  | - | - | - | - | - |  | - | - | - |
| *infγ* | - | - | - | - | - | - | - |  | - | - | - | - | - |  | - | - | - |
| *odc* | 0.006 | 0.004 | - | 0.028 | - | - | - |  | b | b | a | b | b |  | B | AB | A |
| *arg2* | <0.001 | 0.002 | - | <0.001 | - | - | - |  | b | b | b | b | a |  | B | B | A |
| *mtor* | - | - | - | - | - | - | - |  | - | - | - | - | - |  | - | - | - |
| *sod* | - | - | - | - | - | - | - |  | - | - | - | - | - |  | - | - | - |
| *amd1* | - | - | - | - | - | - | - |  | - | - | - | - | - |  | - | - | - |
| *sms* | 0.049 | <0.001 | - | <0.001 | - | - | - |  | ab | a | ab | b | ab |  | B | A | AB |
| *dnmt1* | 0.003 | 0.012 | - | - | - | - | - |  | b | ab | a | b | b |  | B | B | A |
| *dnmt3α* | - | - | - | - | - | - | - |  | - | - | - | - | - |  | - | - | - |
| *dnmt3β* | - | - | - | - | - | - | - |  | - | - | - | - | - |  | - | - | - |
| *ido2* | < 0.001 | <0.001 | <0.001 | <0.001 | <0.001 | <0.001 | <0.001 |  | b | b | b | b | a |  | B | B | A |
| *afmid1* | - | - | - | - | - | - | - |  | - | - | - | - | - |  | - | - | - |

|  | AA × Stimuli | | | | | | | | | | | | | | |  | AA × Time | | | | | | | | | |  | Stimulo x Time | | | | | |
| --- | --- | --- | --- | --- | --- | --- | --- | --- | --- | --- | --- | --- | --- | --- | --- | --- | --- | --- | --- | --- | --- | --- | --- | --- | --- | --- | --- | --- | --- | --- | --- | --- | --- |
|  | L-15 | | | M1x | | | M2x | | | T1x | | | T2x | | |  | L-15 | | M1x | | M2x | | T1x | | T2x | |  | Ø | | LPS*Phdp* | | *Phdp*UV | |
|  | Ø | LPS*Phdp* | *Phdp*UV | Ø | LPS*Phdp* | *Phdp*UV | Ø | LPS*Phdp* | *Phdp*UV | Ø | LPS*Phdp* | *Phdp*UV | Ø | LPS*Phdp* | *Phdp*UV |  | 4h | 24h | 4h | 24h | 4h | 24h | 4h | 24h | 4h | 24h |  | 4h | 24h | 4h | 24h | 4h | 24h |
| *il1β* | - | - | - | - | - | - | - | - | - | - | - | - | - | - | - |  | - | - | - | - | - | - | - | - | - | - |  | - | - | - | - | - | - |
| *il6* | - | - | - | - | - | - | - | - | - | - | - | - | - | - | - |  | - | - | - | - | - | - | - | - | - | - |  | - | - | - | - | - | - |
| *il10* | - | - | - | - | - | - | - | - | - | - | - | - | - | - | - |  | - | - | - | - | - | - | - | - | - | - |  | - | - | - | - | - | - |
| *il13r* | - | - | - | - | - | - | - | - | - | - | - | - | - | - | - |  | - | - | - | - | - | - | - | - | - | - |  | - | - | - | - | - | - |
| *tnfα* | - | - | b | - | - | b | B | B | aA | - | - | b | - | - | ab |  | - | - | - | - | - | - | - | - | - | - |  | - | - | - | - | - | - |
| *cox2* | - | - | - | - | - | - | - | - | - | - | - | - | - | - | - |  | - | - | - | - | - | - | - | - | - | - |  | - | - | - | - | - | - |
| *infγ* | - | - | - | - | - | - | - | - | - | - | - | - | - | - | - |  | - | - | - | - | - | - | - | - | - | - |  | - | - | - | - | - | - |
| *odc* | - | - | b | - | - | b | B | AB | aA | - | - | b | - | - | b |  | - | - | - | - | - | - | - | - | - | - |  | - | - | - | - | - | - |
| *arg2* | - | - | b | - | - | b | - | - | b | - | - | b | B | B | aA |  | - | - | - | - | - | - | - | - | - | - |  | - | - | - | - | - | - |
| *mtor* | - | - | - | - | - | - | - | - | - | - | - | - | - | - | - |  | - | - | - | - | - | - | - | - | - | - |  | - | - | - | - | - | - |
| *sod* | - | - | - | - | - | - | - | - | - | - | - | - | - | - | - |  | - | - | - | - | - | - | - | - | - | - |  | - | - | - | - | - | - |
| *amd1* | - | - | - | - | - | - | - | - | - | - | - | - | - | - | - |  | - | - | - | - | - | - | - | - | - | - |  | - | - | - | - | - | - |
| *sms* | - | b | - | B | aA | B | B | aA | B | - | b | - | - | b | - |  | - | - | - | - | - | - | - | - | - | - |  | - | - | - | - | - | - |
| *dnmt1* | - | - | - | - | - | - | - | - | - | - | - | - | - | - | - |  | - | - | - | - | - | - | - | - | - | - |  | - | - | - | - | - | - |
| *dnmt3α* | - | - | - | - | - | - | - | - | - | - | - | - | - | - | - |  | - | - | - | - | - | - | - | - | - | - |  | - | - | - | - | - | - |
| *dnmt3β* | - | - | - | - | - | - | - | - | - | - | - | - | - | - | - |  | - | - | - | - | - | - | - | - | - | - |  | - | - | - | - | - | - |
| *ido2* | - | - | b | - | - | b | - | - | b | - | - | b | B | B | aA |  | - | b | - | b | - | b | - | b | * | a |  | - | B | - | B | * | A |
| *afmid1* | - | - | - | - | - | - | - | - | - | - | - | - | - | - | - |  | - | - | - | - | - | - | - | - | - | - |  | - | - | - | - | - | - |

Values are presented as means ± SD (n=6). P-values from Multifactorial ANOVA (p ≤0.05). If interaction was significant, Tukey post hoc test was used to identify differences in the experimental treatments. Different lowercase letters stand for significant differences among amino acids treatments while capital letters indicate differences among stimulus. Asterisk indicate significant differences between times.

**Table S2**. Quantitative expression of immune-related gene of head-kidney leucocytes subjected to the experimental conditions for 1, 3 and 6 h.

| Parameters | | L-15 | | | | | | | | | | | | | | | | | | | | | | |
| --- | --- | --- | --- | --- | --- | --- | --- | --- | --- | --- | --- | --- | --- | --- | --- | --- | --- | --- | --- | --- | --- | --- | --- | --- |
|  |  | Ø | | | | | | | | | | |  | AIP56 | | | | | | | | | | |
|  |  | 1h | | |  | 3h | | |  | 6h | | |  | 1h | | |  | 3h | | |  | 6h | | |
| *casp3* | Normalized mRNA expression | 0.00001 | ± | 0.00001 |  | 0.00007 | ± | 0.00015 | B | 0.00001 | ± | 0.00001 |  | 0.00002 | ± | 0.00003 | # | 0.00238 | ± | 0.00165^aA^ |  | 0.00061 | ± | 0.00047*# |
| *casp8* |  | 0.00031 | ± | 0.00021 |  | 0.01211 | ± | 0.00831 |  | 0.00411 | ± | 0.00780 |  | 0.00061 | ± | 0.00030 |  | 0.00858 | ± | 0.00775 |  | 0.00044 | ± | 0.00058^b^ |
| *casp9* |  | 0.00299 | ± | 0.00232 |  | 0.01845 | ± | 0.01620 |  | 0.01031 | ± | 0.01680 |  | 0.05333 | ± | 0.11269 |  | 0.01804 | ± | 0.01898 |  | 0.00141 | ± | 0.00100 |
| *mtor* |  | 0.00001 | ± | 0.00001 |  | 0.00007 | ± | 0.00006 |  | 0.00001 | ± | 0.00001 |  | 0.00000 | ± | 0.00000 |  | 0.00011 | ± | 0.00011 |  | 0.00000 | ± | 0.00000 |
| *il1β* |  | 0.00015 | ± | 0.00021 |  | 0.00041 | ± | 0.00041 |  | 0.00006 | ± | 0.00005 |  | 0.00035 | ± | 0.00040 |  | 0.00005 | ± | 0.00009 |  | 0.00001 | ± | 0.00001 |
| *il8* |  | 0.02987 | ± | 0.02755 |  | 0.04727 | ± | 0.05597 |  | 0.05101 | ± | 0.09300 |  | 0.09483 | ± | 0.11281 |  | 0.01058 | ± | 0.01797 |  | 0.12785 | ± | 0.12814 |
| *tnfa* |  | 0.00463 | ± | 0.00741 |  | 0.02108 | ± | 0.01205 |  | 0.00277 | ± | 0.00306 |  | 0.04711 | ± | 0.09096 |  | 0.01010 | ± | 0.00998 |  | 0.00365 | ± | 0.00306 |
| *nf-ҡb* |  | 0.00014 | ± | 0.00016 |  | 0.00116 | ± | 0.00101 |  | 0.00002 | ± | 0.00003 |  | 0.00007 | ± | 0.00007 |  | 0.00001 | ± | 0.00002 |  | 0.00005 | ± | 0.00004 |
| *p65* |  | 0.00024 | ± | 0.00038 |  | 0.00030 | ± | 0.00026 |  | 0.00022 | ± | 0.00033 |  | 0.00001 | ± | 0.00001 |  | 0.00008 | ± | 0.00008 |  | 0.00000 | ± | 0.00000 |
| *stat3* |  | 0.00004 | ± | 0.00005 |  | 0.00014 | ± | 0.00011 |  | 0.00001 | ± | 0.00001 |  | 0.00005 | ± | 0.00004 |  | 0.00005 | ± | 0.00008 |  | 0.00002 | ± | 0.00001 |
| *sms* |  | 0.00028 | ± | 0.00059 |  | 0.00080 | ± | 0.00078 |  | 0.00000 | ± | 0.00000 |  | 0.00003 | ± | 0.00002 |  | 0.00015 | ± | 0.00020 |  | 0.00002 | ± | 0.00002 |
| *ido2* |  | 0.00309 | ± | 0.00375 |  | 0.00013 | ± | 0.00012 |  | 0.00289 | ± | 0.00575 |  | 0.00000 | ± | 0.00000 |  | 0.00082 | ± | 0.00168 |  | 0.00013 | ± | 0.00010 |
| *afmid1* |  | 0.00001 | ± | 0.00001 |  | 0.00083 | ± | 0.00066 |  | 0.00161 | ± | 0.00193 |  | 0.00001 | ± | 0.00000 |  | 0.00042 | ± | 0.00047 |  | 0.00002 | ± | 0.00003 |
| *amd1* |  | 0.00005 | ± | 0.00007 |  | 0.00142 | ± | 0.00097 |  | 0.00001 | ± | 0.00001 |  | 0.00006 | ± | 0.00004 |  | 0.00103 | ± | 0.00122 |  | 0.00003 | ± | 0.00003 |
| *dnmt1* |  | 0.00001 | ± | 0.00001 |  | 0.00061 | ± | 0.00094 |  | 0.00000 | ± | 0.00000 |  | 0.00002 | ± | 0.00001 |  | 0.00058 | ± | 0.00088 |  | 0.00003 | ± | 0.00004 |
| *dnmt3α* |  | 0.00008 | ± | 0.00007 |  | 0.00008 | ± | 0.00012 |  | 0.00001 | ± | 0.00002 |  | 0.00015 | ± | 0.00011 |  | 0.00001 | ± | 0.00001 |  | 0.00004 | ± | 0.00001 |
| *dnmt3β* |  | 0.00001 | ± | 0.00000 |  | 0.00078 | ± | 0.00063 |  | 0.00000 | ± | 0.00000 |  | 0.00001 | ± | 0.00000 |  | 0.00039 | ± | 0.00066 |  | 0.00002 | ± | 0.00002 |

| Parameters | | M1x | | | | | | | | | | | | | | | | | | | | | | |
| --- | --- | --- | --- | --- | --- | --- | --- | --- | --- | --- | --- | --- | --- | --- | --- | --- | --- | --- | --- | --- | --- | --- | --- | --- |
|  |  | Ø | | | | | | | | | | |  | AIP56 | | | | | | | | | | |
|  |  | 1h | | |  | 3h | | |  | 6h | | |  | 1h | | |  | 3h | | |  | 6h | | |
| *casp3* | Normalized mRNA expression | 0.00007 | ± | 0.00013 |  | 0.00076 | ± | 0.00111 |  | 0.00001 | ± | 0.00001 |  | 0.00004 | ± | 0.00002 |  | 0.00035 | ± | 0.00037^b^ |  | 0.00004 | ± | 0.00008 |
| *casp8* |  | 0.00027 | ± | 0.00038 |  | 0.00835 | ± | 0.00522 |  | 0.00047 | ± | 0.00055 |  | 0.00080 | ± | 0.00070 |  | 0.00690 | ± | 0.00635 |  | 0.00109 | ± | 0.00185^b^ |
| *casp9* |  | 0.00494 | ± | 0.00896 |  | 0.01415 | ± | 0.01127 |  | 0.00080 | ± | 0.00062 |  | 0.00140 | ± | 0.00094 |  | 0.01220 | ± | 0.01374 |  | 0.00410 | ± | 0.00363 |
| *mtor* |  | 0.00001 | ± | 0.00001 |  | 0.00013 | ± | 0.00012 |  | 0.00000 | ± | 0.00000 |  | 0.00002 | ± | 0.00003 |  | 0.00007 | ± | 0.00008 |  | 0.00001 | ± | 0.00002 |
| *il1β* |  | 0.00010 | ± | 0.00009 |  | 0.00043 | ± | 0.00034 |  | 0.00027 | ± | 0.00020 |  | 0.00105 | ± | 0.00070 |  | 0.00050 | ± | 0.00024 |  | 0.00020 | ± | 0.00033 |
| *il8* |  | 0.04674 | ± | 0.08311 |  | 0.01780 | ± | 0.01998 |  | 0.04522 | ± | 0.03117 |  | 0.03690 | ± | 0.02800 |  | 0.02425 | ± | 0.02109 |  | 0.01841 | ± | 0.03386 |
| *tnfa* |  | 0.00784 | ± | 0.01191 |  | 0.01169 | ± | 0.00667 |  | 0.00577 | ± | 0.00660 |  | 0.00429 | ± | 0.00545 |  | 0.00895 | ± | 0.00607 |  | 0.00199 | ± | 0.00183 |
| *nf-ҡb* |  | 0.00039 | ± | 0.00052 |  | 0.00328 | ± | 0.00380 |  | 0.00060 | ± | 0.00080 |  | 0.00014 | ± | 0.00013 |  | 0.00114 | ± | 0.00060 |  | 0.00009 | ± | 0.00015 |
| *p65* |  | 0.00016 | ± | 0.00013 |  | 0.00049 | ± | 0.00051 |  | 0.00001 | ± | 0.00001 |  | 0.00016 | ± | 0.00013 |  | 0.00018 | ± | 0.00017 |  | 0.00008 | ± | 0.00008 |
| *stat3* |  | 0.00002 | ± | 0.00001 |  | 0.00036 | ± | 0.00037 |  | 0.00002 | ± | 0.00002 |  | 0.00004 | ± | 0.00003 |  | 0.00010 | ± | 0.00007 |  | 0.00001 | ± | 0.00001 |
| *sms* |  | 0.00010 | ± | 0.00020 |  | 0.00035 | ± | 0.00025 |  | 0.00001 | ± | 0.00001 |  | 0.00006 | ± | 0.00007 |  | 0.00044 | ± | 0.00041 |  | 0.00008 | ± | 0.00007 |
| *ido2* |  | 0.00932 | ± | 0.02005 |  | 0.00025 | ± | 0.00046 |  | 0.00000 | ± | 0.00000 |  | 0.00000 | ± | 0.00000 |  | 0.00006 | ± | 0.00007 |  | 0.00000 | ± | 0.00000 |
| *afmid1* |  | 0.00035 | ± | 0.00068 |  | 0.00074 | ± | 0.00017 |  | 0.00001 | ± | 0.00000 |  | 0.00004 | ± | 0.00004 |  | 0.00054 | ± | 0.00066 |  | 0.00028 | ± | 0.00043 |
| *amd1* |  | 0.00027 | ± | 0.00054 |  | 0.00050 | ± | 0.00044 |  | 0.00002 | ± | 0.00002 |  | 0.00029 | ± | 0.00049 |  | 0.00117 | ± | 0.00149 |  | 0.00005 | ± | 0.00007 |
| *dnmt1* |  | 0.00019 | ± | 0.00038 |  | 0.00057 | ± | 0.00065 |  | 0.00011 | ± | 0.00013 |  | 0.00047 | ± | 0.00099 |  | 0.00080 | ± | 0.00083 |  | 0.00068 | ± | 0.00082 |
| *dnmt3α* |  | 0.00016 | ± | 0.00025 |  | 0.00088 | ± | 0.00168 |  | 0.00301 | ± | 0.00391 |  | 0.00083 | ± | 0.00165 |  | 0.00005 | ± | 0.00005 |  | 0.00002 | ± | 0.00004 |
| *dnmt3β* |  | 0.00007 | ± | 0.00013 |  | 0.00040 | ± | 0.00043 |  | 0.00002 | ± | 0.00003 |  | 0.00002 | ± | 0.00003 |  | 0.00042 | ± | 0.00046 |  | 0.00007 | ± | 0.00011 |

| Parameters | | M2x | | | | | | | | | | | | | | | | | | | | | |  |
| --- | --- | --- | --- | --- | --- | --- | --- | --- | --- | --- | --- | --- | --- | --- | --- | --- | --- | --- | --- | --- | --- | --- | --- | --- |
|  |  | Ø | | | | | | | | | | |  | AIP56 | | | | | | | | | | |
|  |  | 1h | | |  | 3h | | |  | 6h | | |  | 1h | | |  | 3h | | |  | 6h | | |
| *casp3* | Normalized mRNA expression | 0.00001 | ± | 0.00001 |  | 0.00004 | ± | 0.00003 |  | 0.00001 | ± | 0.00001 |  | 0.00009 | ± | 0.00018 |  | 0.00011 | ± | 0.00016^b^ |  | 0.00002 | ± | 0.00001 |
| *casp8* |  | 0.00005 | ± | 0.00003 |  | 0.00127 | ± | 0.00117 |  | 0.00240 | ± | 0.00448 |  | 0.00459 | ± | 0.00911 |  | 0.00304 | ± | 0.00345 |  | 0.00069 | ± | 0.00054^b^ |
| *casp9* |  | 0.00086 | ± | 0.00143 |  | 0.00449 | ± | 0.00459 |  | 0.00096 | ± | 0.00081 |  | 0.00027 | ± | 0.00044 |  | 0.00753 | ± | 0.00674 |  | 0.00221 | ± | 0.00127 |
| *mtor* |  | 0.00000 | ± | 0.00000 |  | 0.00002 | ± | 0.00002 |  | 0.00000 | ± | 0.00000 |  | 0.00001 | ± | 0.00002 |  | 0.00002 | ± | 0.00002 |  | 0.00000 | ± | 0.00000 |
| *il1β* |  | 0.00007 | ± | 0.00007 |  | 0.00032 | ± | 0.00036 |  | 0.00020 | ± | 0.00033 |  | 0.00040 | ± | 0.00019 |  | 0.00069 | ± | 0.00058 |  | 0.00042 | ± | 0.00042 |
| *il8* |  | 0.00018 | ± | 0.00012 |  | 0.18353 | ± | 0.23754 |  | 0.07255 | ± | 0.11222 |  | 0.06611 | ± | 0.06706 |  | 0.18081 | ± | 0.21206 |  | 0.02178 | ± | 0.02827 |
| *tnfa* |  | 0.00011 | ± | 0.00008 |  | 0.00772 | ± | 0.00885 |  | 0.00131 | ± | 0.00222 |  | 0.00234 | ± | 0.00488 |  | 0.01227 | ± | 0.00909 |  | 0.00249 | ± | 0.00166 |
| *nf-ҡb* |  | 0.00058 | ± | 0.00066 |  | 0.00069 | ± | 0.00066 |  | 0.00013 | ± | 0.00015 |  | 0.00000 | ± | 0.00000 |  | 0.00046 | ± | 0.00050 |  | 0.00012 | ± | 0.00011 |
| *p65* |  | 0.00003 | ± | 0.00006 |  | 0.00003 | ± | 0.00003 |  | 0.00005 | ± | 0.00005 |  | 0.00014 | ± | 0.00017 |  | 0.00015 | ± | 0.00015 |  | 0.00004 | ± | 0.00006 |
| *stat3* |  | 0.00000 | ± | 0.00000 |  | 0.00030 | ± | 0.00049 |  | 0.00002 | ± | 0.00004 |  | 0.00001 | ± | 0.00002 |  | 0.00008 | ± | 0.00008 |  | 0.00003 | ± | 0.00001 |
| *sms* |  | 0.00000 | ± | 0.00000 |  | 0.00010 | ± | 0.00010 |  | 0.00001 | ± | 0.00001 |  | 0.00034 | ± | 0.00035 |  | 0.00017 | ± | 0.00018 |  | 0.00004 | ± | 0.00004 |
| *ido2* |  | 0.00040 | ± | 0.00090 |  | 0.00003 | ± | 0.00003 |  | 0.00001 | ± | 0.00001 |  | 0.00014 | ± | 0.00013 |  | 0.00001 | ± | 0.00002 |  | 0.00196 | ± | 0.00339 |
| *afmid1* |  | 0.00000 | ± | 0.00000 |  | 0.00008 | ± | 0.00010 |  | 0.00001 | ± | 0.00001 |  | 0.00029 | ± | 0.00040 |  | 0.00025 | ± | 0.00024 |  | 0.00004 | ± | 0.00003 |
| *amd1* |  | 0.00000 | ± | 0.00000 |  | 0.00022 | ± | 0.00019 |  | 0.00003 | ± | 0.00004 |  | 0.00012 | ± | 0.00023 |  | 0.00020 | ± | 0.00021 |  | 0.00008 | ± | 0.00006 |
| *dnmt1* |  | 0.00000 | ± | 0.00000 |  | 0.00006 | ± | 0.00007 |  | 0.00000 | ± | 0.00001 |  | 0.00026 | ± | 0.00048 |  | 0.00016 | ± | 0.00023 |  | 0.00042 | ± | 0.00042 |
| *dnmt3α* |  | 0.00164 | ± | 0.00125 |  | 0.00010 | ± | 0.00016 |  | 0.00001 | ± | 0.00002 |  | 0.00018 | ± | 0.00019 |  | 0.00275 | ± | 0.00531 |  | 0.00003 | ± | 0.00003 |
| *dnmt3β* |  | 0.00000 | ± | 0.00000 |  | 0.00006 | ± | 0.00006 |  | 0.00000 | ± | 0.00001 |  | 0.00025 | ± | 0.00056 |  | 0.00012 | ± | 0.00019 |  | 0.00004 | ± | 0.00002 |

| Parameters | | T1x | | | | | | | | | | | | | | | | | | | | | | |
| --- | --- | --- | --- | --- | --- | --- | --- | --- | --- | --- | --- | --- | --- | --- | --- | --- | --- | --- | --- | --- | --- | --- | --- | --- |
|  |  | Ø | | | | | | | | | | |  | AIP56 | | | | | | | | | | |
|  |  | 1h | | |  | 3h | | |  | 6h | | |  | 1h | | |  | 3h | | |  | 6h | | |
| *casp3* | Normalized mRNA expression | 0.00000 | ± | 0.00000 |  | 0.00001 | ± | 0.00001 |  | 0.00000 | ± | 0.00000 |  | 0.00004 | ± | 0.00003 |  | 0.00048 | ± | 0.00055^b^ |  | 0.00182 | ± | 0.00157 |
| *casp8* |  | 0.00009 | ± | 0.00007 |  | 0.00761 | ± | 0.01514 |  | 0.00012 | ± | 0.00009 |  | 0.00073 | ± | 0.00079^#^ |  | 0.01854 | ± | 0.03065^#^ |  | 0.07214 | ± | 0.05812^a*^ |
| *casp9* |  | 0.00007 | ± | 0.00000 |  | 0.00082 | ± | 0.00064 |  | 0.00090 | ± | 0.00061 |  | 0.00961 | ± | 0.00402 |  | 0.00569 | ± | 0.00424 |  | 0.00132 | ± | 0.00082 |
| *mtor* |  | 0.00000 | ± | 0.00000 |  | 0.00000 | ± | 0.00000 |  | 0.00000 | ± | 0.00000 |  | 0.00003 | ± | 0.00003 |  | 0.00002 | ± | 0.00001 |  | 0.00006 | ± | 0.00008 |
| *il1β* |  | 0.00001 | ± | 0.00000 |  | 0.00010 | ± | 0.00012 |  | 0.00001 | ± | 0.00000 |  | 0.00000 | ± | 0.00000 |  | 0.00001 | ± | 0.00001 |  | 0.00004 | ± | 0.00005 |
| *il8* |  | 0.00022 | ± | 0.00016 |  | 0.00173 | ± | 0.00229 |  | 0.02951 | ± | 0.02534 |  | 0.00015 | ± | 0.00016 |  | 0.00241 | ± | 0.00266 |  | 0.00184 | ± | 0.00212 |
| *tnfa* |  | 0.04029 | ± | 0.04022 |  | 0.00198 | ± | 0.00235 |  | 0.00280 | ± | 0.00245 |  | 0.00035 | ± | 0.00025 |  | 0.00360 | ± | 0.00333 |  | 0.00106 | ± | 0.00112 |
| *nf-ҡb* |  | 0.00000 | ± | 0.00000 |  | 0.00005 | ± | 0.00002 |  | 0.00010 | ± | 0.00009 |  | 0.00002 | ± | 0.00003 |  | 0.00035 | ± | 0.00033 |  | 0.00001 | ± | 0.00001 |
| *p65* |  | 0.00000 | ± | 0.00000 |  | 0.00001 | ± | 0.00002 |  | 0.00000 | ± | 0.00000 |  | 0.00006 | ± | 0.00008 |  | 0.00010 | ± | 0.00012 |  | 0.00001 | ± | 0.00001 |
| *stat3* |  | 0.00000 | ± | 0.00000 |  | 0.00001 | ± | 0.00000 |  | 0.00002 | ± | 0.00002 |  | 0.00000 | ± | 0.00000 |  | 0.00004 | ± | 0.00005 |  | 0.00001 | ± | 0.00001 |
| *sms* |  | 0.00000 | ± | 0.00000 |  | 0.00006 | ± | 0.00001 |  | 0.00002 | ± | 0.00002 |  | 0.00004 | ± | 0.00006 |  | 0.00008 | ± | 0.00009 |  | 0.00000 | ± | 0.00000 |
| *ido2* |  | 0.00001 | ± | 0.00000 |  | 0.00003 | ± | 0.00000 |  | 0.00000 | ± | 0.00000 |  | 0.00106 | ± | 0.00180 |  | 0.00002 | ± | 0.00002 |  | 0.00110 | ± | 0.00219 |
| *afmid1* |  | 0.00000 | ± | 0.00000 |  | 0.00004 | ± | 0.00002 |  | 0.00000 | ± | 0.00000 |  | 0.00001 | ± | 0.00001 |  | 0.00007 | ± | 0.00009 |  | 0.00000 | ± | 0.00001 |
| *amd1* |  | 0.00000 | ± | 0.00000 |  | 0.00033 | ± | 0.00042 |  | 0.00000 | ± | 0.00000 |  | 0.00001 | ± | 0.00002 |  | 0.00048 | ± | 0.00056 |  | 0.00001 | ± | 0.00001 |
| *dnmt1* |  | 0.00000 | ± | 0.00000 |  | 0.00001 | ± | 0.00002 |  | 0.00001 | ± | 0.00001 |  | 0.00003 | ± | 0.00005 |  | 0.00010 | ± | 0.00012 |  | 0.00000 | ± | 0.00000 |
| *dnmt3α* |  | 0.00000 | ± | 0.00000 |  | 0.00001 | ± | 0.00001 |  | 0.00005 | ± | 0.00006 |  | 0.00150 | ± | 0.00260 |  | 0.00003 | ± | 0.00005 |  | 0.00003 | ± | 0.00003 |
| *dnmt3β* |  | 0.00000 | ± | 0.00000 |  | 0.00002 | ± | 0.00002 |  | 0.00000 | ± | 0.00000 |  | 0.00000 | ± | 0.00001 |  | 0.00007 | ± | 0.00006 |  | 0.00000 | ± | 0.00000 |

| Parameters | | T2x | | | | | | | | | | | | | | | | | | | | | | |
| --- | --- | --- | --- | --- | --- | --- | --- | --- | --- | --- | --- | --- | --- | --- | --- | --- | --- | --- | --- | --- | --- | --- | --- | --- |
|  |  | Ø | | | | | | | | | | |  | AIP56 | | | | | | | | | | |
|  |  | 1h | | |  | 3h | | |  | 6h | | |  | 1h | | |  | 3h | | |  | 6h | | |
| *casp3* | Normalized mRNA expression | 0.00001 | ± | 0.00001 |  | 0.00001 | ± | 0.00001 |  | 0.00026 | ± | 0.00043 |  | 0.00105 | ± | 0.00209 |  | 0.00064 | ± | 0.00023^b^ |  | 0.00191 | ± | 0.00093 |
| *casp8* |  | 0.00009 | ± | 0.00010 |  | 0.00166 | ± | 0.00356 |  | 0.00197 | ± | 0.00193 |  | 0.01048 | ± | 0.01782 |  | 0.01649 | ± | 0.02481 |  | 0.01664 | ± | 0.01203^b^ |
| *casp9* |  | 0.00088 | ± | 0.00047 |  | 0.00617 | ± | 0.01206 |  | 0.02985 | ± | 0.03565 |  | 0.03323 | ± | 0.06068 |  | 0.00308 | ± | 0.00447 |  | 0.01042 | ± | 0.01267 |
| *mtor* |  | 0.00000 | ± | 0.00000 |  | 0.00001 | ± | 0.00001 |  | 0.00010 | ± | 0.00015 |  | 0.00001 | ± | 0.00001 |  | 0.00003 | ± | 0.00002 |  | 0.00027 | ± | 0.00015 |
| *il1β* |  | 0.00002 | ± | 0.00002 |  | 0.00002 | ± | 0.00002 |  | 0.00001 | ± | 0.00001 |  | 0.00005 | ± | 0.00005 |  | 0.00006 | ± | 0.00006 |  | 0.00002 | ± | 0.00001 |
| *il8* |  | 0.00491 | ± | 0.00654 |  | 0.00241 | ± | 0.00363 |  | 0.04707 | ± | 0.08017 |  | 0.00417 | ± | 0.00489 |  | 0.00709 | ± | 0.00764 |  | 0.00175 | ± | 0.00065 |
| *tnfa* |  | 0.00063 | ± | 0.00081 |  | 0.00184 | ± | 0.00267 |  | 0.01442 | ± | 0.02293 |  | 0.00163 | ± | 0.00178 |  | 0.00333 | ± | 0.00282 |  | 0.00078 | ± | 0.00098 |
| *nf-ҡb* |  | 0.00002 | ± | 0.00002 |  | 0.00009 | ± | 0.00016 |  | 0.00113 | ± | 0.00154 |  | 0.00001 | ± | 0.00001 |  | 0.00001 | ± | 0.00001 |  | 0.00000 | ± | 0.00000 |
| *p65* |  | 0.00001 | ± | 0.00001 |  | 0.00001 | ± | 0.00002 |  | 0.00007 | ± | 0.00008 |  | 0.00000 | ± | 0.00000 |  | 0.00000 | ± | 0.00000 |  | 0.00000 | ± | 0.00000 |
| *stat3* |  | 0.00000 | ± | 0.00000 |  | 0.00002 | ± | 0.00004 |  | 0.00014 | ± | 0.00020 |  | 0.00002 | ± | 0.00003 |  | 0.00002 | ± | 0.00002 |  | 0.00007 | ± | 0.00007 |
| *sms* |  | 0.00000 | ± | 0.00000 |  | 0.00003 | ± | 0.00003 |  | 0.00066 | ± | 0.00111 |  | 0.00006 | ± | 0.00009 |  | 0.00028 | ± | 0.00049 |  | 0.00000 | ± | 0.00000 |
| *ido2* |  | 0.00369 | ± | 0.00516 |  | 0.00045 | ± | 0.00059 |  | 0.00011 | ± | 0.00017 |  | 0.00003 | ± | 0.00005 |  | 0.00038 | ± | 0.00023 |  | 0.00004 | ± | 0.00003 |
| *afmid1* |  | 0.00017 | ± | 0.00033 |  | 0.00002 | ± | 0.00003 |  | 0.00067 | ± | 0.00086 |  | 0.00005 | ± | 0.00005 |  | 0.00013 | ± | 0.00010 |  | 0.00000 | ± | 0.00000 |
| *amd1* |  | 0.00001 | ± | 0.00001 |  | 0.00002 | ± | 0.00004 |  | 0.00050 | ± | 0.00081 |  | 0.00005 | ± | 0.00006 |  | 0.00001 | ± | 0.00001 |  | 0.00001 | ± | 0.00001 |
| *dnmt1* |  | 0.00001 | ± | 0.00001 |  | 0.00003 | ± | 0.00004 |  | 0.00072 | ± | 0.00119 |  | 0.00007 | ± | 0.00007 |  | 0.00001 | ± | 0.00000 |  | 0.00001 | ± | 0.00001 |
| *dnmt3α* |  | 0.00003 | ± | 0.00005 |  | 0.00001 | ± | 0.00002 |  | 0.00003 | ± | 0.00006 |  | 0.00006 | ± | 0.00008 |  | 0.00001 | ± | 0.00001 |  | 0.00003 | ± | 0.00001 |
| *dnmt3β* |  | 0.00000 | ± | 0.00000 |  | 0.00018 | ± | 0.00036 |  | 0.00064 | ± | 0.00106 |  | 0.00010 | ± | 0.00018 |  | 0.00016 | ± | 0.00009 |  | 0.00000 | ± | 0.00000 |

| Multifactorial ANOVA | | |  |  |  |  |  |  |  |  |  |  |  |  |  |  |  |
| --- | --- | --- | --- | --- | --- | --- | --- | --- | --- | --- | --- | --- | --- | --- | --- | --- | --- |
| Parameters | AA | Stimuli | Time | AA × Stimuli | AA × Time | Stimuli × Time | AA × Stimuli × Time |  | AA | | | | |  | Time | | |
|  |  |  |  |  |  |  |  |  |  |  |  |  |  |  |  |  |  |
|  |  |  |  |  |  |  |  |  | L-15 | M1x | M2x | T1x | T2x |  | 1h | 3h | 6h |
| *casp3* | - | <0.001 | 0.030 | 0.002 | - | - | 0.011 |  | - | - | - | - | - |  | # | * | *# |
| *casp8* | 0.006 | 0.002 | - | 0.002 | 0.019 | - | 0.011 |  | b | b | b | a | ab |  | - | - | - |
| *casp9* | - | - | - | - | - | - | - |  | - | - | - | - | - |  | - | - | - |
| *mtor* | - | - | 0.001 | - | <0.001 | - | - |  | - | - | - | - | - |  | # | * | * |
| *il1β* | <0.001 | 0.029 | - | 0.027 | - | 0.032 | - |  | bc | a | ab | c | c |  | - | - | - |
| *il8* | 0.002 | - | - | - | 0.015 | - | - |  | ab | ab | a | b | b |  | - | - | - |
| *tnfα* | - | - | - | - | - | - | - |  | - | - | - | - | - |  | - | - | - |
| *nf-ҡb* | 0.001 | 0.021 | 0.007 | - | 0.012 | - | - |  | ab | b | ab | a | c |  | # | * | # |
| *p65* | 0.011 | - | - | - | - | - | - |  | ab | a | abc | c | c |  | - | - | - |
| *stat3* | - | - | <0.001 | - | 0.019 | - | - |  | - | - | - | - | - |  | # | * | # |
| *sms* | - | - | 0.004 | 0.024 | - | - | - |  | - | - | - | - | - |  | # | * | # |
| *ido2* | - | - | - | - | - | - | - |  | - | - | - | - | - |  | - | - | - |
| *afmid1* | 0.025 | - | - | - | - | - | - |  | a | ab | ab | b | ab |  | - | - | - |
| *amd1* | - | - | <0.001 | - | 0.004 | - | - |  | - | - | - | - | - |  | # | * | # |
| *dnmt1* | 0.038 | - | - | - | - | - | - |  | ab | a | ab | b | ab |  | - | - | - |
| *dnmt3a* | - | - | - | - | - | - | - |  | - | - | - | - | - |  | - | - | - |
| *dnmt3b* | - | - | 0.007 | - | 0.026 | - | - |  | - | - | - | - | - |  | # | * | # |

| Parameters | AA × Stimuli | | | | | | | | | |  | AA × Time | | | | | | | | | | | | | | |  | Stimuli × Time | | | | | |
| --- | --- | --- | --- | --- | --- | --- | --- | --- | --- | --- | --- | --- | --- | --- | --- | --- | --- | --- | --- | --- | --- | --- | --- | --- | --- | --- | --- | --- | --- | --- | --- | --- | --- |
|  | L-15 | | M1x | | M2x | | T1x | | T2x | |  | L-15 | | | M1x | | | M2x | | | T1x | | | T2x | | |  | Ø | | | AIP56 | | |
|  | Ø | AIP56 | Ø | AIP56 | Ø | AIP56 | Ø | AIP56 | Ø | AIP56 |  | 1h | 3h | 6h | 1h | 3h | 6h | 1h | 3h | 6h | 1h | 3h | 6h | 1h | 3h | 6h |  | 1h | 3h | 6h | 1h | 3h | 6h |
| *casp 3* | B | aA |  | b |  | b |  | ab | B | aA |  | - | - | - | - | - | - | - | - | - | - | - | - | - | - | - |  | - | - | - | - | - | - |
| *casp8* |  | b |  | b |  | ab |  | a |  | ab |  |  |  | b |  |  | b |  |  | b | # | *# | a* |  |  | ab |  | - | - | - | - | - | - |
| *casp9* | - | - | - | - | - | - | - | - | - | - |  | - | - | - | - | - | - | - | - | - | - | - | - | - | - | - |  | - | - | - | - | - | - |
| *mtor* | - | - | - | - | - | - | - | - | - | - |  | # | * | b*# | # | * | b# |  |  | b |  |  | b | # | # | a* |  | - | - | - | - | - | - |
| *il1β* |  | b |  | a |  | a |  | b |  | b |  | - | - | - | - | - | - | - | - | - | - | - | - | - | - | - |  | B |  |  | A |  |  |
| *il8* | - | - | - | - | - | - | - | - | - | - |  | - | b | - | - | b | - | # | a* | *# | - | b | - | - | b | - |  | - | - | - | - | - | - |
| *tnfa* | - | - | - | - | - | - | - | - | - | - |  | - | - | - | - | - | - | - | - | - | - | - | - | - | - | - |  | - | - | - | - | - | - |
| *nf-kb* | - | - | - | - | - | - | - | - | - | - |  |  | b |  | # | a* | # |  | b |  |  | b |  |  | b |  |  | - | - | - | - | - | - |
| *p65* | - | - | - | - | - | - | - | - | - | - |  | - | - | - | - | - | - | - | - | - | - | - | - | - | - | - |  | - | - | - | - | - | - |
| *stat3* | - | - | - | - | - | - | - | - | - | - |  |  | ab |  | # | a* | *# |  | ab |  |  | b |  |  | b |  |  | - | - | - | - | - | - |
| *sms* | a |  | ab |  | ab |  | ab |  | b |  |  | - | - | - | - | - | - | - | - | - | - | - | - | - | - | - |  | - | - | - | - | - | - |
| *ido2* | - | - | - | - | - | - | - | - | - | - |  | - | - | - | - | - | - | - | - | - | - | - | - | - | - | - |  | - | - | - | - | - | - |
| *afmid1* | - | - | - | - | - | - | - | - | - | - |  | - | - | - | - | - | - | - | - | - | - | - | - | - | - | - |  | - | - | - | - | - | - |
| *amd1* | - | - | - | - | - | - | - | - | - | - |  | # | a* | # |  | a |  |  | b |  |  | ab |  |  | b |  |  | - | - | - | - | - | - |
| *dnmt1* | - | - | - | - | - | - | - | - | - | - |  | - | - | - | - | - | - | - | - | - | - | - | - | - | - | - |  | - | - | - | - | - | - |
| *dnmt3a* | - | - | - | - | - | - | - | - | - | - |  | - | - | - | - | - | - | - | - | - | - | - | - | - | - | - |  | - | - | - | - | - | - |
| *dnmt3b* | - | - | - | - | - | - | - | - | - | - |  | # | a* | # |  | ab |  |  | ab |  |  | b |  |  | ab |  |  | - | - | - | - | - | - |

Values are presented as means ± SD (n=6). P-values from Multifactorial ANOVA (p ≤0.05). If interaction was significant, Tukey post hoc test was used to identify differences in the experimental treatments. Different lowercase letters stand for significant differences among amino acids treatments while capital letters indicate differences among stimulus. Different symbols stand for significant differences between times.
